# Tailoring a Lead-Free Organic–Inorganic Halobismuthate for Large Piezoelectric Effect

**DOI:** 10.1021/jacs.5c15484

**Published:** 2025-11-25

**Authors:** Esther Y.H. Hung, Benjamin M. Gallant, Robert Harniman, Jakob Möbs, Santanu Saha, Khaled Kaja, Charles Godfrey, Shrestha Banerjee, Nikolaos Famakidis, Harish Bhaskaran, Marina R. Filip, Paolo Radaelli, Nakita K. Noel, Dominik J. Kubicki, Harry C. Sansom, Henry J. Snaith

**Affiliations:** 1 Clarendon Laboratory, Department of Physics, 6396 University of Oxford, Oxford OX1 3PU, United Kingdom; 2 School of Chemistry, 1724 University of Birmingham, Edgbaston, Birmingham B15 2TT, United Kingdom; 3 Department of Solution-Processing of Hybrid Materials and Devices, Helmholtz-Zentrum Berlin für Materialien und Energie GmbH, Berlin 12489, Germany; 4 School of Chemistry, 1980 University of Bristol, Bristol BS8 1TS, United Kingdom; 5 Institute for Inorganic and Analytical Chemistry, Justus-Liebig-University Gießen, Gießen 35392, Germany; 6 27025 Institut de Recherche sur les Céramiques (IRCER), UMR CNRS 7315-Université de Limoges, Limoges 87068, France; 7 Bruker Nano Surfaces and Metrology, Karlsruhe 76187, Germany; 8 Department of Materials, 6396 University of Oxford, Oxford OX1 3PH, United Kingdom

## Abstract

Molecular piezoelectrics are a potentially disruptive technology, enabling a new generation of self-powered electronics that are flexible, high performing, and inherently low in toxicity. Although significant efforts have been made toward understanding their structural design by targeted manipulation of phase transition behavior, the resulting achievable piezoresponse has remained limited. In this work, we use a low-symmetry, zero-dimensional (0D) inorganic framework alongside a carefully selected ‘quasi-spherical’ organic cation to manipulate organic–inorganic interactions and thus form the hybrid, piezoelectric material [(CH_3_)_3_NCH_2_I]_3_Bi_2_I_9_. Using variable–temperature single crystal X-ray diffraction and solid-state nuclear magnetic resonance spectroscopy, we demonstrate that this material simultaneously exhibits an order–disorder and displacive symmetry-breaking phase transition. This phase transition is mediated by halogen bonding between the organic and inorganic frameworks and results in a large piezoelectric response, *d*_33_ = 161.5 pm/V. This value represents a 4-fold improvement on previously reported halobismuthate piezoelectrics and is comparable to those of commercial inorganic piezoelectrics, thus offering a new pathway toward low-cost, low-toxicity mechanical energy harvesting and actuating devices.

## Graphical abstract

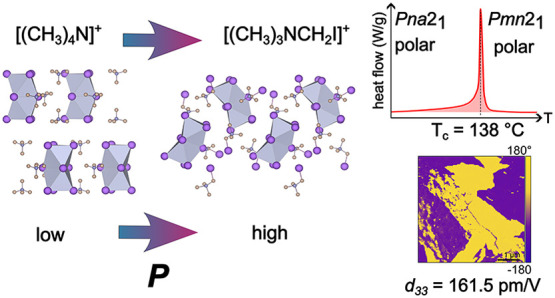

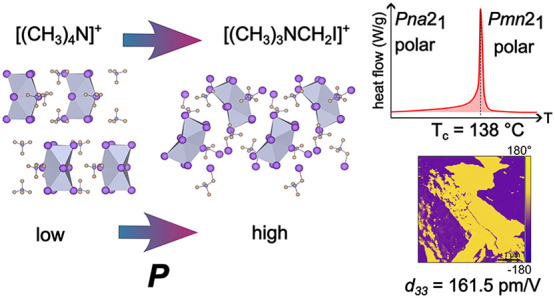

## Introduction

Owing to their lack of inversion symmetry, piezoelectric materials can generate electrical polarization in response to mechanical stress, and vice versa. Among these, ferroelectrics tend to be the best performing as they exhibit spontaneous polarization, which can be switched by an external electric field to produce a stronger piezoelectric response, described by the piezoelectric coefficients *d*_*ij*_, where *i* indicates the direction of resulting polarization and *j* the direction and type of mechanical stress. High-performance ferroelectrics such as lead zirconium titanate (PZT) ceramics (*d*_*ij*_ ∼ 200–600 pC/N) have been used extensively in devices such as sensors, actuators, and energy harvesting systems.^1,2^ However, as they consist of more than 60% lead by weight,^3^ the use of these ceramics raises significant environmental and regulatory concerns as global restrictions on toxic substances continue to tighten.^4^ Despite substantial research efforts on alternative lead-free ceramics such as BaTiO_3_ and NaNbO_3_, their piezoelectric performance remains limited (*d*_*ij*_ ∼ 50–200 pC/N).^5,6^ Moreover, ceramic oxides are highly brittle and require rigorous compositional control throughout production, alongside expensive processing under high temperature and pressure.^7^ Despite their high *d*_*ij*_s, these conditions strongly hinder their suitability for emerging technologies that demand flexibility and low-cost manufacturing such as wearable sensors and flexible devices.

Consequently, significant effort has focused on developing a new generation of low-toxicity materials that combine high piezoelectric coefficients, facile fabrication, and mechanical flexibility. Among these, hybrid organic–inorganic metal halide perovskites and their derivatives have garnered considerable attention. Hybrid perovskites have risen to prominence in photovoltaic and optoelectronic applications owing to their exceptional tolerance to defects and impressive optoelectronic properties.^8^ While there have been notable successes in applying hybrid perovskite-related compounds to piezoelectrics, including [(CH_3_)_3_NCH_2_Cl]­MnCl_3_ (*d*_33_ = 185 pC/N)^9^ and [(CH_3_)_3_NCH_2_F]_*x*_[(CH_3_)_3_NCH_2_Cl]_1–*x*_CdCl_3_ (*d*_33_ = 1540 pC/N)^10^ (Table S1), these advances have still relied on toxic and heavy metals, such as Mn and Cd, highlighting the continuing need for low-toxicity ferroelectrics.^11–13^

To address these limitations requires careful choice of inorganic framework, since this forms an important backbone for developing polar and noncentrosymmetric structures. In this respect, reduced-dimensionality structures (0D, 1D or 2D) not only exhibit reduced symmetry, but also broaden the diversity in choice of organic cations. This structural tunability has enabled the development of various strategies to engineer hybrid metal halide ferroelectrics with symmetry-breaking phase transitions at critical temperatures (*T*_c_) above room temperature, inducing spontaneous polarization in the materials under application-relevant conditions.

Such phase transitions can be classified as either order–disorder or displacive transitions. One design principle used to manipulate order–disorder transitions is the so-called “quasi-spherical theory”.^14,15^ In this approach, structurally symmetric organic cations are chemically modified to break their symmetry. Typically, these organic cations exhibit high-symmetry, rapid, disordered motion—typically whole molecule tumbling—above *T*_c_, which is lost upon cooling as the asymmetric organic cations become static in the structure, inducing net polarization. Hence these cations are known as ‘quasi-spherical’. Another design strategy is to control the nature and strength of organic–inorganic interactions in the low temperature phase by deliberately selecting two components that bind together in such a way that enforces noncentrosymmetry in their relative positions, and thus a net polarization. At elevated temperature, these interactions break, and the organic and inorganic components are released to occupy a higher symmetry arrangement, resulting in a displacive-type phase transition.^16,17^ For example, Hua et al. recently investigated the role of halogen bonding between the organic and inorganic components of [(CH_3_)_3_NCH_2_X]­PbI_3_ (X = Cl, Br, I) and found that the strongest halogen-halide interaction occurred for X = I (C–I···I^–^), leading to a maximum *T*_c_ = 39 °C.^18^ The authors suggested that this correlation is due to an increase in the onset temperature of organic cation tumbling in the material. Similar approaches in Cd- and Mn-based materials have been reported using weaker C–Cl···Cl^–^ and C–Br···Br^–^ interactions,^19,20^ and the resulting enhanced piezoresponse tentatively attributed to manipulation of an order–disorder phase transition, although further experimental support is needed to provide clarity.

Inspired by these ideas, we select quasi-spherical [(CH_3_)_3_NCH_2_I]^+^ (trimethyl­(iodomethyl)­ammonium = TMIM^+^) as the cation and combine it with a nontoxic, 0D iododobismuthate anionic framework to synthesize (TMIM)_3_Bi_2_I_9_, which remains polar until its decomposition temperature of 250 °C and exhibits the highest piezoelectric coefficient ever reported for a halobismuthate, to the best of our knowledge, when processed as a thin film. We synthesize (TMIM)_3_Bi_2_I_9_ not only via thin film solution processing, but also as single crystals by a solution crystallization method and as microcrystalline powders via mechanosynthesis, demonstrating the broad and facile processability of this material. By conducting in-depth, variable–temperature structural analysis that combines synchrotron-based single crystal X-ray diffraction (SCXRD) with high-field solid-state nuclear magnetic resonance (NMR) spectroscopy and density functional theory (DFT) calculations, we establish the structural origins of the phase transition behavior of (TMIM)_3_Bi_2_I_9_. In particular, we detail experimentally how halogen bonding between the organic TMIM^+^ cation and inorganic [Bi_2_I_9_]^3–^ dimer can be utilized to manipulate phase transition behavior and enhance polarization in the desirable piezoelectric phase; a strategy that is applicable across all such hybrid organic–inorganic piezoelectrics.

## Results and Discussion

### Inducing Noncentrosymmetry for High Piezoresponse

Dark red single crystals of (TMIM)_3_Bi_2_I_9_ are synthesized by slow cooling of an aqueous solution of HI (see the Supporting Information Experimental Section). Structural analyses of single crystal X-ray diffraction (SCXRD) data (Figure 1a) show that (TMIM)_3_Bi_2_I_9_ crystallizes in the orthorhombic polar space group *Pna*2_1_ at 20 °C. Notably, this is a noncentrosymmetric space group, satisfying the most fundamental structural criterion for a piezoelectric material. In comparison to the polar phase of its organohalide-free counterpart, [(CH_3_)_4_N]_3_Bi_2_I_9_ (Figure 1b), we observe several features desirable for high piezoresponse in (TMIM)_3_Bi_2_I_9_. First, the [Bi_2_I_9_]^3+^ dimers are oriented at ±26.5° with respect to the longest crystallographic *a* axis, toward the *c* axis, and nearly perpendicular ±88.8° to the *b* axis (Figure 1a). This degree of asymmetry in the dimer is unusual for A_3_B_2_X_9_ structures, where the [Bi_2_X_9_]^3+^ (X = I, Br, Cl) dimers are typically aligned close to or parallel to the long crystallographic axes.^21–25^ This tilting in (TMIM)_3_Bi_2_I_9_ leads to a reduced-symmetry doubling of the unit cell compared with [(CH_3_)_4_N]_3_Bi_2_I_9_.^26^ Additionally, we observe a pronounced distortion within the [Bi_2_X_9_]^3+^ dimer of (TMIM)_3_Bi_2_I_9_, originating from asymmetry in the Bi1–I_b1_–Bi2 bond distances for one of the bridging iodides (I_b1_): the Bi1–I_b1_ distance is 3.14 Å, while the Bi2–I_b1_ distance is 3.29 Å. The [Bi_2_I_9_]^3+^ dimer in (TMIM)_3_Bi_2_I_9_ also lacks rotational symmetry about the Bi···Bi axis (Figure 1a). In contrast, the [Bi_2_X_9_]^3+^ dimers found in [(CH_3_)_4_N]_3_Bi_2_I_9_ are nearly symmetric in the face-sharing plane of μ_2_-bridging iodides, as well as exhibiting both 3-fold rotational (*C*_3_) and vertical mirror plane (σ_v_) site symmetry (Figure 1b). Importantly, the significant asymmetry in (TMIM)_3_Bi_2_I_9_ is expected to result in a greater polarization than in the higher-symmetry [(CH_3_)_4_N]_3_Bi_2_I_9_, ultimately improving the piezoelectric response.

**Figure 1:**
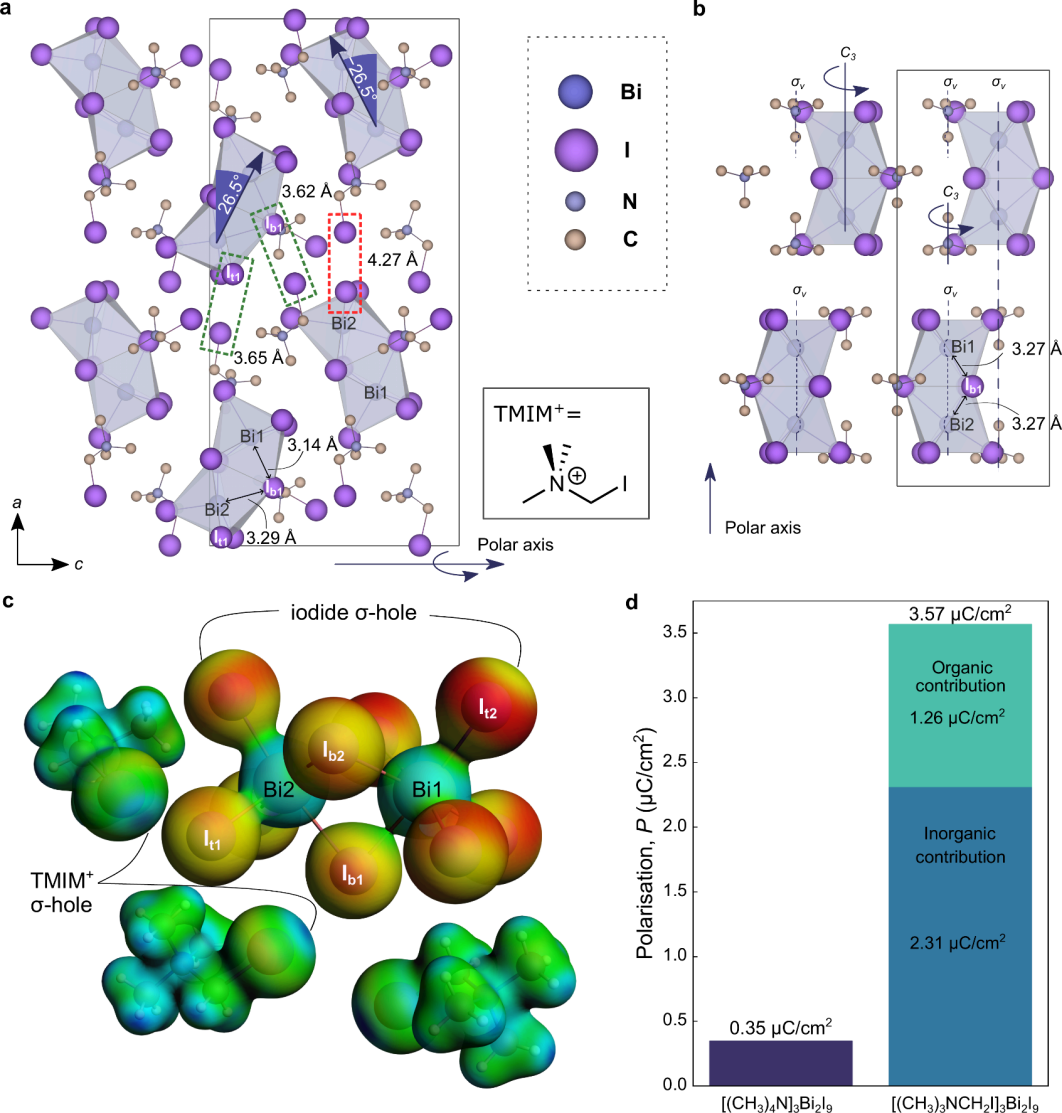
Inducing noncentrosymmetry for high piezoresponse. (a) Room temperature crystal structure of [(CH_3_)_3_NCH_2_I]_3_Bi_2_I_9_. Lower right: chemical structure of [(CH_3_)_3_NCH_2_I]^+^ or TMIM^+^. (b) Crystal structure of [(CH_3_)_4_N]_3_Bi_2_I_9_ reproduced from ICSD code 170840. Hydrogen atoms are omitted for clarity. (c) Electrostatic potential (ESP) surface of (TMIM)_3_Bi_2_I_9_, where the color scheme represents electrostatic potential from red (more negative) to blue (more positive), and the sigma hole on the iodine of TMIM^+^ is shown to be oriented toward the inorganic framework. (d) Calculated net polarization for [(CH_3_)_4_N]_3_Bi_2_I_9_ and [(CH_3_)_3_NCH_2_I]_3_Bi_2_I_9_.

To establish this, we adopt a point charge model for polarization that considers only the inorganic components and apply it to (TMIM)_3_Bi_2_I_9_ and [(CH_3_)_4_N]_3_Bi_2_I_9_. This analysis reveals that the tilted and distorted dimers of (TMIM)_3_Bi_2_I_9_ result in an order-of-magnitude increase in polarization along the polar 2_1_ screw-axis, compared to that along the 3_1_ axis of [(CH_3_)_4_N]_3_Bi_2_I_9_ (Note S1 and Figure S1). Moreover, the ±26.5° tilting of the dimer in (TMIM)_3_Bi_2_I_9_ leads to increased void volume between the dimers along its polar *c* axis compared to the polar (long) axis in [(CH_3_)_4_N]_3_Bi_2_I_9_, which may allow for increased displacement of ions and improve polarization.^27^

Since the inorganic [Bi_2_I_9_]^3–^ dimer is compositionally unchanged in (TMIM)_3_Bi_2_I_9_ compared to [(CH_3_)_4_N]_3_Bi_2_I_9_, the distortion of the inorganic framework in (TMIM)_3_Bi_2_I_9_ must arise from the asymmetric, polar TMIM^+^ cations and their interaction with the [Bi_2_I_9_]^3–^ dimers. In the room temperature structure, determined by SCXRD, the TMIM^+^ cations exhibit well-defined positions with pronounced asymmetry around each dimer. Specifically, all three organic cations associated with each dimer are positioned such that their iodomethyl groups orient toward different I^–^ ions within the same BiI_6_ octahedron (Bi2, in Figure 1a). This asymmetric arrangement contrasts with the high-symmetry positioning of [(CH_3_)_4_N]^+^ cations in [(CH_3_)_4_N]_3_Bi_2_I_9_, where their contribution to the overall polarization across the unit cell is nearly equal in magnitude and antiparallel to the contribution from the [Bi_2_I_9_]^3+^ dimers, resulting in near-to-total canceling out of the net polarization (Note S1). Therefore, the ordered TMIM^+^ cations disrupt this cancellation and contribute to a higher net polarization in (TMIM)_3_Bi_2_I_9_.

Importantly, we identify that the I···I^–^ distances between two of these cations and their nearest neighbor iodides in the [Bi_2_I_9_]^3–^ dimer (green dashed boxes, Figure 1a), 3.62 Å and 3.65 Å, are substantially less than the sum of the van der Waals radius of iodine and the Shannon ionic radius of iodide (4.18 Å). This suggests the presence of a bonding interaction between the organic and inorganic components; one between a TMIM^+^ cation and a bridging iodide (I_b1_) and another between a second TMIM^+^ cation and a terminal iodide (I_t1_). The C–I···I^–^ bond angles corresponding to these interactions (172.0° and 163.2°) are consistent with the presence of halogen bonds, in which iodide serves as the electron donating species.

To investigate the presence of these interactions, we calculate the electrostatic potential (ESP) surface surrounding the [(CH_3_)_3_NCH_2_I]^+^ cation and observe a significant positive electrostatic well localized on the iodine atom and antiparallel to the C–I bond (Figure S2). This electrostatic potential motif is commonly described as a “σ-hole” in the electron density surrounding the iodine and is essential for effective halogen bonding.^28–30^ The positively charged quaternary ammonium substituent on the iodomethyl group of TMIM^+^ acts as a highly effective electron-withdrawing group, directing positive charge into the σ-hole. We next calculate the ESP across the (TMIM)_3_Bi_2_I_9_ structure (Figure 1c). For the two short I···I^–^ distances identified, the σ-hole of each TMIM^+^ is directed toward the adjacent iodide, consistent with halogen bond formation. However, the ESP map shows only a small amount of shared electron density between the two atoms (Figure S3). This suggests a high degree of ionicity in these halogen bonds, as expected for a halogen-halide interaction between two ions.

The ESP map also reveals a second type of σ-hole in the (TMIM)_3_Bi_2_I_9_ structure, located on the iodide ions, and particularly pronounced on terminal iodides not involved in halogen bonding, e.g. I_t2_. This feature originates from the covalent nature of the Bi–I bonds, which leads to regions of relative electropositivity on the iodide antiparallel to the Bi–I bond axis. The presence of this σ-hole is important in rationalizing the observed geometry in the room temperature structure of (TMIM)_3_Bi_2_I_9_. For instance, the near-perpendicular Bi2–I_t1_···I bond angle of 93.1° arises to minimize electrostatic repulsion between the σ-holes of TMIM^+^ and I_t1_ (Figure 1c). The iodide σ-hole formation similarly governs the orientation of the halogen bond to I_b1_, where a Bi2–I_b1_···I bond angle of 94.1° is observed. Critically, this geometry leads to a nearly linear Bi1–I_b1_···I bond angle of 176.3°, which appears to unfavorably orient the TMIM^+^ σ-hole directly toward an I_b1_ σ-hole. However, analysis of the electron density distribution across the nonhalogen-bonded I_b2_, suggests that σ-hole formation on μ_2_-bridging iodides is not as pronounced as on terminal iodides due to the orthogonal arrangement of the two Bi–I bonds (Figure 1c). Thus, the I_b1_ halogen bond orientation may not be as energetically unfavorable as expected.

The influence of the halogen bond on the Bi1–I_b1_ bond distance is also particularly striking. As noted above, the Bi1–I_b1_ bond is unexpectedly shorter than the Bi2–I_b1_ bond (Figure 1a). Typically, the donation of electron density from I_b1_ toward the σ-hole of TMIM^+^ might be expected to weaken and lengthen the Bi1–I_b1_ bond. Instead, the observed bond shortening suggests that the donation of electron density originates from a molecular orbital of the [Bi_2_I_9_]^3–^ dimer which is antibonding in nature; the depopulation of this orbital serves to strengthen the Bi1–I_b1_ bond. Furthermore, the energetic benefit of this bond strengthening likely contributes to the otherwise unfavorable head-on orientation of the iodide (I_b1_) and TMIM^+^ σ-holes.

Crucially, it is this halogen bond-induced shortening of the Bi1–I_eq1_ bond that drives the desymmetrization of the [Bi_2_I_9_]^3–^ dimer and is thus responsible for its large polarization, as identified above. Similarly, the orientation of this halogen bond (176.3° and 94.1° to the Bi1–I_b1_ and Bi2–I_b1_ bonds, respectively)—determined by the interaction between the σ-holes of TMIM^+^ and I_b1_—anchors the TMIM^+^ cations in their highly asymmetric arrangement around the dimer, highlighted above in contrast to the symmetric positioning of the organics in [(CH_3_)_4_N]_3_Bi_2_I_9_. Taken together with the asymmetric tilting of the dimers away from the crystallographic axes, these halogen bond-induced structural phenomena are expected to contribute to significant polarization across the overall (TMIM)_3_Bi_2_I_9_ structure and thus enhance its piezoelectric response.

To verify these structural findings, we calculate the polarization vectors of (TMIM)_3_Bi_2_I_9_ and [(CH_3_)_4_N]_3_Bi_2_I_9_ using the Berry phase approach in plane-wave density functional theory (DFT) (Figure 1d).^31–36^ To isolate the contribution of the tilted and distorted inorganic framework to net polarization from that of the network of TMIM^+^ cations, we first replace the TMIM^+^ cations with [(CH_3_)_4_N]^+^ while preserving the inorganic framework geometry of (TMIM)_3_Bi_2_I_9_. This substitution yields a net polarization of 2.31 μC/cm^2^ along the crystallographic *c* axis. By contrast, conducting the same calculation using the true, undistorted [(CH_3_)_4_N]_3_Bi_2_I_9_ structure yields a significantly lower polarization of 0.35 μC/cm^2^. This 6-fold increase demonstrates the crucial role of the distorted inorganic framework in (TMIM)_3_Bi_2_I_9_ in the overall polarization of the structure. When considering the complete system including the TMIM^+^ cations, the overall polarization for (TMIM)_3_Bi_2_I_9_ is calculated to be 3.57 μC/cm^2^, an order of magnitude higher than that of [(CH_3_)_4_N]_3_Bi_2_I_9_. This increase in polarization is consistent with our point charge model calculations (Note S1), and comparable or larger than that of other organic–inorganic metal halide ferroelectrics.^9,20,37,38^ Additional calculations were performed by replacing TMIM^+^ with Cs^+^, which resulted in a polarization similar to that observed with the high-symmetry, nonpolar [(CH_3_)_4_N]^+^ cation (Table S2).

Crucially, we have demonstrated through both experimental structural analysis and theoretical calculations that introducing intermolecular interactions—in this case halogen bonding—induces structural tilting, distortion and increased asymmetry in (TMIM)_3_Bi_2_I_9_. This leads to a significant enhancement in polarization compared to the system containing nonhalogenated organics, demonstrating the potential to leverage intermolecular interactions for tunable and optimized piezoelectric properties.

### Mechanism of Symmetry Breaking in the Phase Transition

The phase transition behavior of (TMIM)_3_Bi_2_I_9_ at elevated temperature is first studied by conducting differential scanning calorimetry (DSC, Figure 2a). Upon heating, an endothermic event at 139 °C is observed, which is reversible with an exotherm at 137 °C upon cooling, indicating a transition temperature at 138 °C. The sharp, well-defined peaks observed in both heating and cooling cycles suggest that this transition is first-order in nature. The calculated molar enthalpy change (Δ*H*) and molar entropy change (Δ*S*) for this transition are +8.85 kJ mol^–1^ and +21.5 J mol^–1^ K^–1^, respectively. This value of Δ*S* is larger than those reported previously for order–disorder transitions in hybrid organic–inorganic halides (∼5–15 J mol^–1^ K^–1^),^39,40^ and more comparable to molecular ferroelectrics.^41,42^ Using Δ*S* = *R*ln*N* = 21.5 J mol^–1^ K^–1^ (where *R* is the molar gas constant and *N* the ratio of the number of geometrically distinguishable orientations between the two phases) we find *N* = 13, suggesting the onset of high-symmetry orientational reconfiguration upon phase transition.

**Figure 2:**
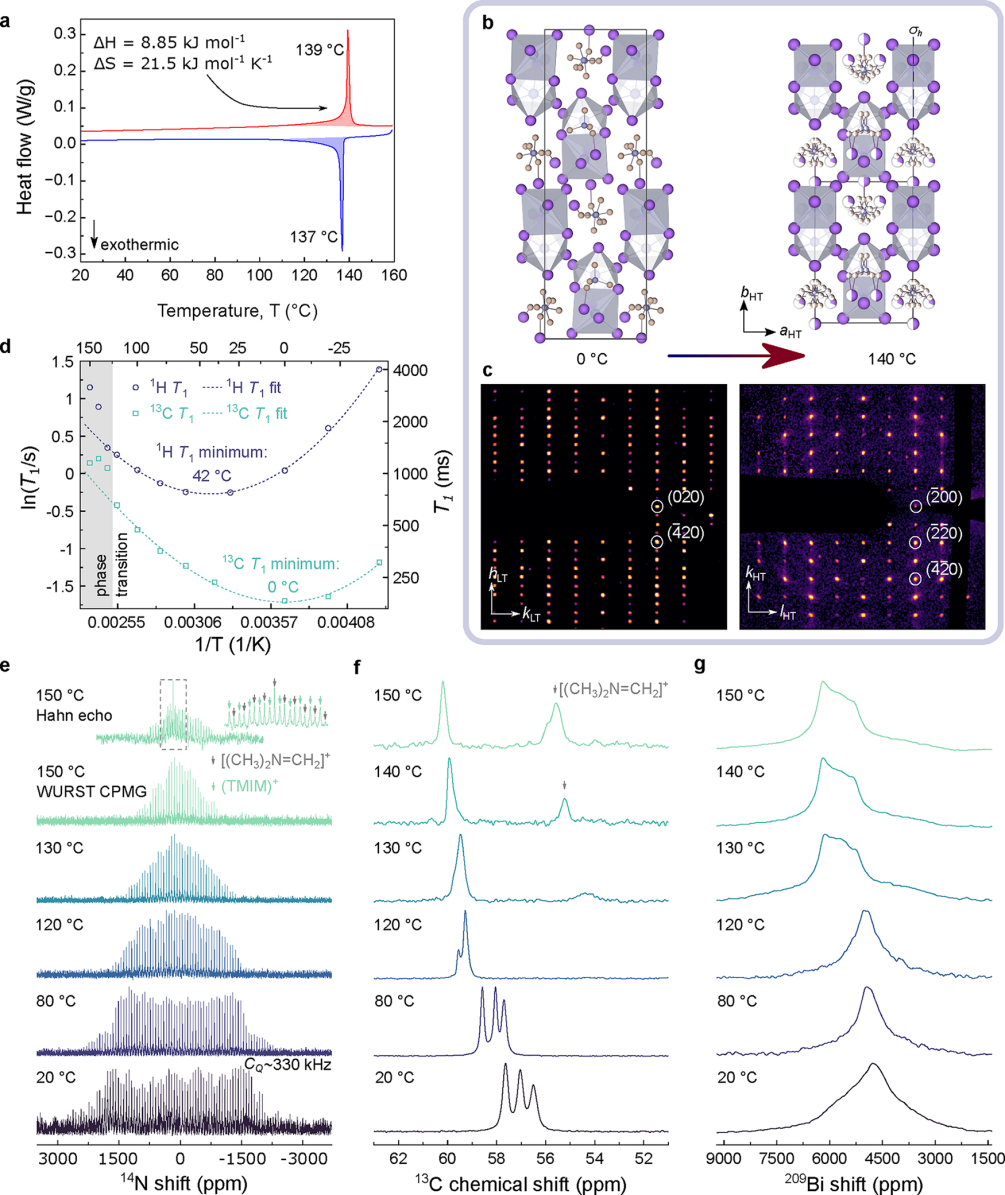
Phase transition behavior. (a) Differential scanning calorimetry (DSC) trace of (TMIM)_3_Bi_2_I_9_, with integrated areas used to calculate molar enthalpy change (Δ*H*) and molar entropy change (Δ*S*) indicated. (b) Crystal structures and (c) (*hk*0) reciprocal space reconstructions of (TMIM)_3_Bi_2_I_9_ at 0 °C (left) and 140 °C (right), respectively. Hydrogen atoms are omitted for clarity. The reciprocal space reconstructions have been plotted on a square root intensity scale to make the diffuse scattering features clearer. (d) Temperature dependence of ^1^H and ^13^C NMR spin–lattice relaxation times, *T*_1_, observed for (TMIM)_3_Bi_2_I_9_. To guide the eye, these data are fitted to quadratic functions, which are extrapolated above the phase transition temperature. Variable–temperature ^14^N (e), ^13^C (f), and ^209^Bi (g) solid-state nuclear magnetic resonance (NMR) spectra of (TMIM)_3_Bi_2_I_9_. WURST CPMG is a method to record wide-line NMR spectra (see the Supporting Information Experimental Section).

We investigate the structural evolution of (TMIM)_3_Bi_2_I_9_ with temperature using variable–temperature (VT) SCXRD (Figure 2b) and identify a polar-to-polar structural phase transition occurring at 140 °C, from the room temperature (RT) *Pna*2_1_ to high temperature (HT) *Pmn*2_1_. This structural transition is associated with a gain of translational symmetry along the *a*_LP_ (*b*_HT_) axes leading to an approximate halving of the unit cell volume (*b*_HT_ = 1/2 *a*_LT_), evident in the (*hk*0)_LT_ and (0*kl*)_HT_ reciprocal space reconstructions, indicated by doubling of the Bragg peak spacing along (*h*00)_LT_ and (0*k*0)_HT_ (Figure 2c). Temperature-induced, rod-like diffuse scattering is also observed in the HT reciprocal space reconstruction, signifying deviations or disorder relative to the average structure derived from long-range Bragg scattering.^43^

Analysis of the crystal structure with increasing temperature shows that the [Bi_2_I_9_]^3–^ dimer increases in symmetry by transitioning from general Wyckoff positions in the LT phase to a special position with σ_*h*_ symmetry in the HT phase. This transition is accompanied by a linear change with temperature in the tilt of the inorganic dimer toward the *a*_LT_ axis from ±26.5 to 26.0° (as visualized in Figure 1a) just before the phase transition; upon the phase transition it discontinuously tilts back to ±26.3° (Figure S4). Furthermore, the positions of the TMIM^+^ cations with respect to the dimer shift by up to 0.74 Å (Figure S5 and Table S3), indicating a displacive transition. The increase in symmetry as a result of this displacive transition halves the net polarization contribution from the inorganic dimer, as calculated usingthe point charge model (Note S1). The retention of dimer tilting in the HT phase indicates that the polar, noncentrosymmetric nature of (TMIM)_3_Bi_2_I_9_ is preserved even if the disordered organic component is ignored.

The TMIM^+^ cations undergo more significant change in the HT phase, transitioning from an ordered and distinct arrangement to a state of 2-fold orientational disorder across a mirror plane (σ_*h*_, Figure 2b). During the peak indexing process for data reduction (via *xia2*),^44^ diffuse scattering is discarded, resulting in a slight loss of electron density in the HT difference Fourier map compared to the RT structure. This leads to less than 100% refined occupancy of the TMIM^+^ cations in the HT phase: one TMIM^+^ is refined to 63% occupancy split across two positions, one refined to 48% occupancy over two positions, and one to 100% occupancy split equally across two positions. The residual electron density map shows weak residual density surrounding these TMIM^+^ cations (Figure S6). The observed diffuse scattering is thus attributed to the dynamic molecular behavior of the organic component. Notably, (TMIM)_3_Bi_2_I_9_ retains a polar and noncentrosymmetric phase up its decomposition temperature (250 °C, Figure S7), demonstrating significant advantage over other hybrid materials for applications across a wide temperature range.^9,18,45^

To gain deeper insight into the phase transition between the two phases of (TMIM)_3_Bi_2_I_9_, variable–temperature multinuclear solid-state nuclear magnetic resonance (VT NMR) spectroscopy is employed. VT NMR is a uniquely powerful tool to investigate the evolution in both local structure and dynamic motion within solid materials. First, we measure the ^1^H and ^13^C spin–lattice relaxation rate time constants (*T*_1_) associated with nuclei in the TMIM^+^ cations as a function of temperature (Figure 2d). The dynamic processes responsible for the *T*_1_ minima observed are discussed in detail in Note S2. Phase transitions lead to discontinuities in the quadratic behavior around *T*_1_ minima. The change in curvature observed at around 130 °C in both ^1^H and ^13^C *T*_1_ suggests a change in the degrees of freedom of TMIM^+^, confirming that the organic cation plays an active role in the phase transition.

To isolate the role of (TMIM)^+^ reorientation from other motions we conduct static VT ^14^N NMR at 20.0 T (Figure 2e). ^14^N is a quadrupolar spin *I =* 1 nucleus and its lineshapes are highly sensitive to local symmetry and dynamics.^46,47^ In [(CH_3_)_3_NCH_2_I]^+^ the inequivalent substituents generate a substantial electric field gradient (EFG) across the ^14^N nucleus, leading to a large quadrupole coupling constant (*C*_Q_), which determines the line width of TMIM^+^ in the absence of reorientation. However, as reported for [CH_3_NH_3_]^+^ and [(NH_2_)_2_CH]^+^ cations in photovoltaic halide perovskites, rapid whole molecule reorientation with correlation times comparable to or shorter than 1/*C*_Q_ act to dynamically average the EFG.^46–49^ In the regime of fast EFG reorientation, the width of the spectral envelope is dictated by the cation’s local environment rather than its intrinsic symmetry, leading to a reduced *C*_Q_ and spectral narrowing. Since the EFG is located on the nitrogen, it can only change due to a whole-body reorientation of the organic cation. The broad (330 kHz) ^14^N signal envelope remains unchanged between RT and 100 °C, and narrows significantly between 120 and 150 °C, coinciding with the phase transition temperature. The 3-fold reduction in line width observed between 120 and 150 °C demonstrates that TMIM^+^ becomes appreciably dynamic at the phase transition temperature, reorienting with correlation times comparable to the inverse of the static *C*_Q_, i.e. on the microsecond time scale. The onset of such motion is consistent with halogen bonds breaking at elevated temperatures, resulting in an order–disorder phase transition.

In order to resolve the individual temperature-dependent behavior of the three structurally inequivalent TMIM^+^ in (TMIM)_3_Bi_2_I_9_, we conduct VT ^1^H–^13^C cross-polarization (CP) magic angle spinning (MAS). Figure 2f shows the ^13^C region corresponding to the iodomethyl group of TMIM^+^. At room temperature, three distinct signals appear reflecting the presence of three inequivalent TMIM^+^ cations in the unit cell. As temperature increases, all three signals shift to higher frequency. By 120 °C, two merge into a single narrow peak (full-width-half-maximum, fwhm = 35 Hz), and by 140 °C all three merge. This result indicates that the three TMIM^+^ cations progressively become magnetically equivalent and indistinguishable with increasing temperature, owing to their tumbling.

To isolate the role of the inorganic sublattice of (TMIM)_3_Bi_2_I_9_ in the phase transition, we perform static ^209^Bi NMR (Figure 2g). ^209^Bi is a quadrupolar nucleus (spin *I* = 9/2), typically leading to large *C*_Q_ values and broad NMR signals.^50^ While we do not observe clearly resolved quadrupolar lineshapes at any temperature, likely due to signal overlap from two structurally inequivalent Bi^3+^ and additional line broadening due to nearby quadrupolar ^127^I nuclei,^51^ we see a substantial change in the ^209^Bi spectrum on crossing the phase transition temperature. This result is consistent with the pronounced reorientation of the [Bi_2_I_9_]^3–^ dimers at the phase transition observed via SCXRD and confirms displacive component of the transformation. Since SCXRD shows neglible changes of the bond lengths and angles within the dimer upon crossing the phase transition, , we attribute the marked change in the ^209^Bi lineshape to the release of halogen bonding interactions between the organic and inorganic components, altering the local environment of the [Bi_2_I_9_]^3–^ dimers.

In both ^14^N MAS (Figure 2e) and ^1^H–^13^C CPMAS NMR spectra recorded at high temperature, a decomposition product is observed forming over the course of several hours of measurement. Using a combination of ^1^H and ^13^C MAS NMR we identify this decomposition pathway as the pyrolysis of (TMIM)^+^ to form iodomethane gas and *N*,*N*-dimethylmethaniminium iodide ([(CH_3_)_2_N = CH_2_]^+^, commonly known as Eschenmoser’s salt) (Figure S27). This result is consistent with that of the original 1971 study by Eschenmoser.^52^ The onset of this decomposition occurs at approximately 130 °C, which should therefore be considered the limiting temperature for long-term thermal stability of (TMIM)_3_Bi_2_I_9_.

Together, the VT SCXRD, ^13^C NMR and ^209^Bi NMR measurements show that a substantial rearrangement of both the inorganic dimers and organic cations, independently and relative to each other, takes place during the phase transition. ^14^N NMR and VT SCXRD, supported by ^13^C NMR and DSC analysis, confirms the simultaneous onset of rapid, microsecond time scale, organic cation tumbling within the structure at the phase transition. Importantly, these findings therefore confirm that symmetry breaking at the phase transition of (TMIM)_3_Bi_2_I_9_ involves contributions from both order–disorder (organic cation tumbling) and displacive (structural rearrangement) components, both enabled by the release of C–I···I^–^ halogen bonds between the organic and inorganic components.

### Piezoelectric Response in (TMIM)_3_Bi_2_I_9_

Having established the origins of symmetry breaking between the two phases of (TMIM)_3_Bi_2_I_9_, we next measure the piezoelectric response at room temperature. Generally, piezoelectric response is comprised of intrinsic (lattice displacement) and extrinsic (movement of domain walls) contributions.^54^ Although accurately distinguishing their relative contributions is experimentally challenging, the intrinsic piezoresponse can be quantified using *in situ* SCXRD under an applied electric field.

To directly measure lattice parameter strain under an applied electric field, we perform *in situ* SCXRD measurements using a method developed by Saunders et al.^55^ Here, DC voltage is applied to a crystal while it is mounted on the diffractometer and held at constant temperature in a cryostream. SCXRD measurements are recorded at different applied electric field strengths, from positive to negative 4 kV/mm across the *c* axis (as determined by crystal morphology simulation)^53^ (Figure 3b). The evolution of refined lattice parameters is shown in Figure 3c. Over this range, the *a* and *b* lattice parameters linearly decrease by 0.01% and 0.007% respectively, while a larger linear increase of the *c* lattice parameter by 0.035% is observed, leading to an overall increase in unit cell volume. The absolute change in individual lattice parameters under applied field (0.001 Å at 1 kV/mm) is comparable to values previously reported for high-performance piezoelectrics PZT and PMN–PT.^56,57^

**Figure 3:**
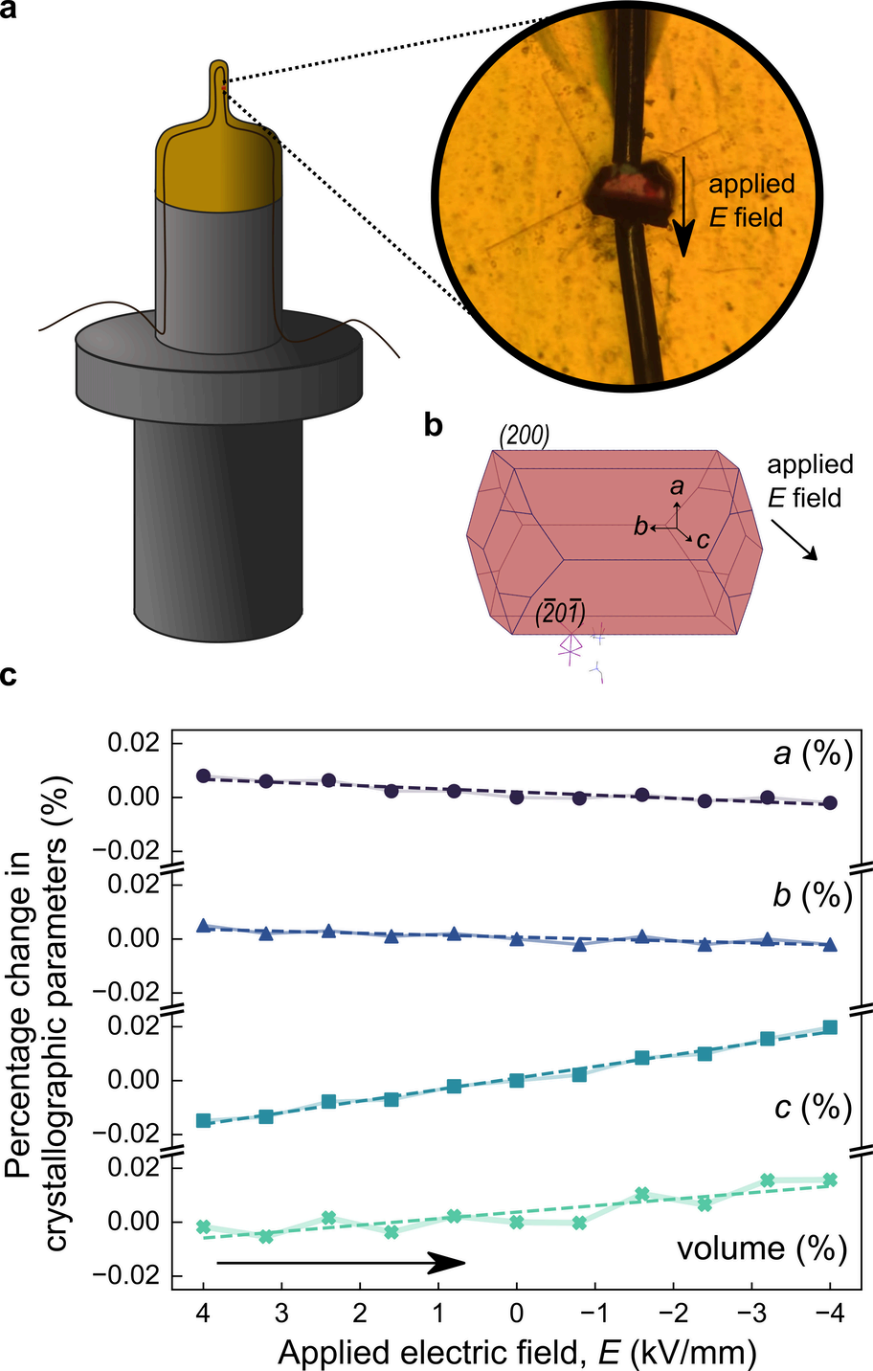
Intrinsic piezoresponse via *in situ* electric field measurements. (a) Left: visualization of the crystal mounted on Kapton, connected to copper electrodes. Right: microscope image of the crystal connected to electrodes. (b) Crystal growth morphology of (TMIM)_3_Bi_2_I_9_ simulated using the Bravais–Friedel–Donnay–Harker (BFDH) method in Mercury.^53^ (c) Percentage change of lattice parameters and unit cell volume with applied electric field *E*, where field is swept from positive to negative. The error bands on each measurement are indicated by the shaded region about each point.

The electrical dependencies can be used to extract information about the intrinsic piezoelectric coefficients;

$$d_{kij}=\frac{\partial \varepsilon_{ij}}{\partial E_{k}}$$

where ε_*ij*_ is the strain tensor relating to the change in the lattice parameters, and *E*_*k*_ are the components of the electric field.

Using the off-field lattice parameters (*a*_0_, *b*_0_, *c*_0_ = 29.9188, 9.8303, 14.1641 Å, respectively) and the fitted linear regression coefficients from Figure 3c, the piezoelectric tensor coefficients *d*_31_, *d*_32_ and *d*_33_ are calculated to be 11.7, 0.7 and −42.8 pC/N respectively. Single crystals frequently contain nanoscale domains that fall below the detection limits of SCXRD, yielding diffraction patterns that represent a coherent average across all domains. Therefore, the derived piezoelectric coefficients from lattice parameter variations reflect domain-averaged values, which are expected to be lower than those of individual domains.

For practical applications such as energy harvesters, sensors and actuators, piezoelectric materials processed into thin films are often preferred.^69^ Therefore, we next process (TMIM)_3_Bi_2_I_9_ into a thin film to investigate its piezoelectric properties using piezoresponse force microscopy (PFM). Figure 4a–c presents the topography, piezoelectric amplitude and phase response, where we identify the presence of piezoelectric domains with 180° phase difference, indicating oppositely oriented polarizations. We observe these domains spanning multiple crystal grains, confirming that the signal originates from the polarization state rather than topographical artifacts.

**Figure 4:**
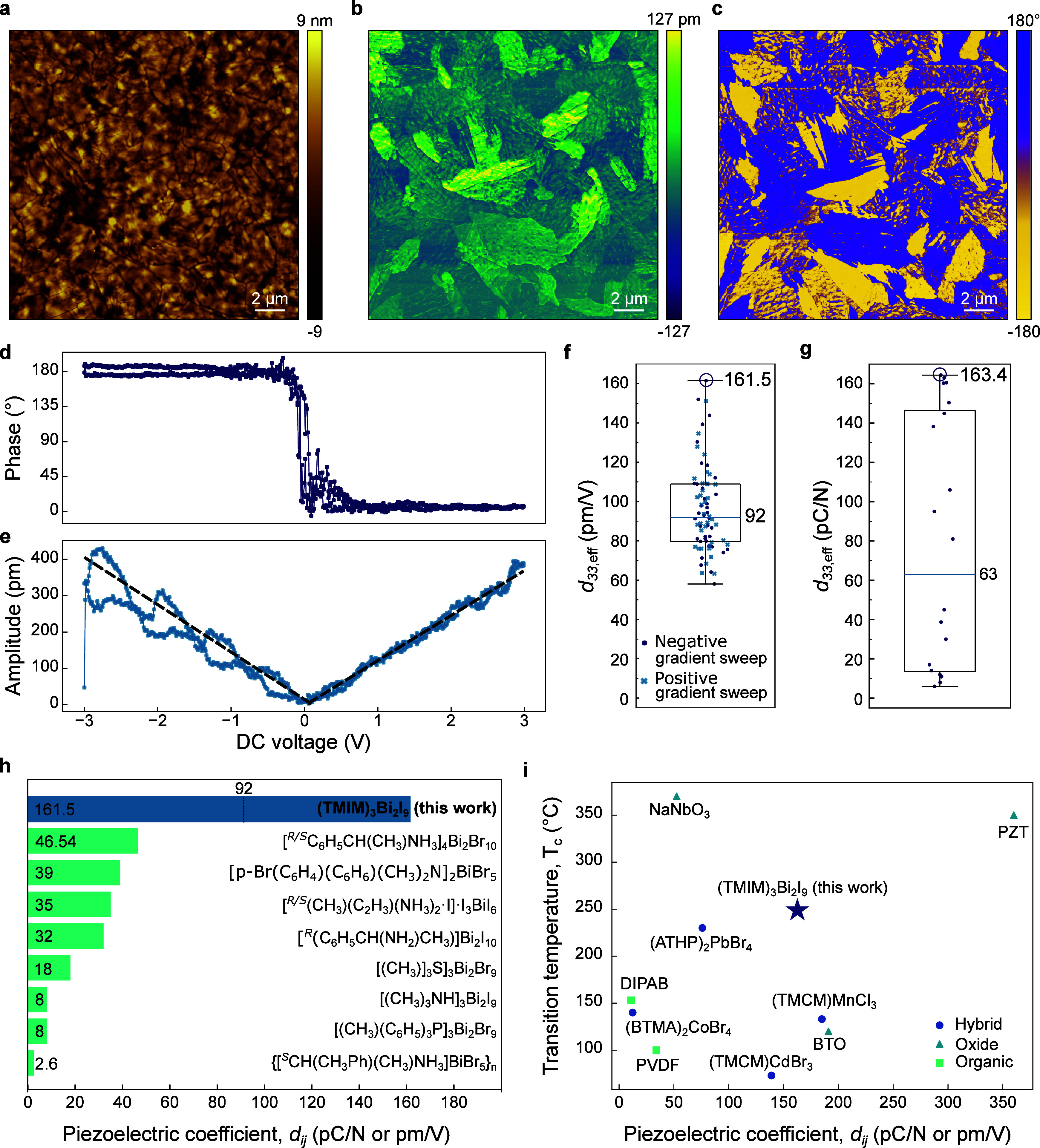
Highest piezoelectric response for a halobismuthate. Piezoresponse force microscopy: topography (a), piezoelectric amplitude (b), and phase images (c). Variable–voltage PFM measurements. Phase–voltage (d) and amplitude–voltage (e) loops, where the dashed line indicates the linear fit used to calculate the piezoelectric coefficient. Box plots showing *d*_33,eff_ values calculated from amplitude–voltage loops via PFM (f) and the Berlincourt method on single crystals (g), respectively, where the whiskers show the entire range of data. Champion values for each measurement are circled. For the piezoelectric coefficients calculated from PFM, the absolute values obtained from the negative and positive gradient are indicated. (h) Comparison of piezoelectric coefficients *d*_*ij*_ with previously reported piezoelectric halobismuthates,^45,58–64^ where the median value from PFM measurements is indicated for (TMIM)_3_Bi_2_I_9_. (i) Comparison of phase transition temperature and piezoelectric coefficients with previously reported piezoelectric materials, where the decomposition temperature is used when no piezo-to-nonpiezoelectric transition temperature is reported.^1,5,6,9,19,65–68^ The full data are shown in Table S1.

To investigate the polarization switching behavior and piezoresponse, we measure the amplitude and phase response at different locations on the thin film while sweeping the cantilever tip DC voltage between −3 and 3 V, with a superimposed AC voltage. Representative phase-bias and amplitude-bias loops are shown in Figure 4d,e, respectively, and show clear 180° polarization switching. The effective piezoelectric coefficient (*d*_33,eff_) can be determined using the gradient of the linear fit of the amplitude-bias loops. A boxplot of calculated values from different sample locations is shown in Figure 4f, yielding a median *d*_33,eff_ of 92.0 pm/V and a peak value of 161.5 pm/V. We note that measurements in regions with a higher calculated *d*_33,eff_ exhibit more pronounced fluctuations around the linear fit (Figure 4e). This is attributed to the stronger electromechanical responsivity of these regions, which amplifies the effects of local material inhomogeneities and instrumental noise. The distribution of the piezoelectric coefficients can be attributed to the multioriented and multidomain nature of the thin film.

We further confirm the piezoelectric behavior via the Berlincourt method on single crystals, which uses the direct piezoelectric effect.^70^ As the crystals do not exhibit a natural face corresponding to a (*00l*) plane, piezoelectric measurements were measured over multiple orientations of the crystal. Therefore, the measured *d*_33,eff_ in Figure 4g correspond to a distribution over the natural crystal faces. This yielded an average *d*_33,eff_ of 63 pC/N, with a peak value of 163.4 pC/N_,_ in agreement with the peak value obtained from PFM (we note that pC/N is equivalent to the unit pm/V). The values obtained from the Berlincourt method and PFM are much greater than that obtained from the lattice strain measurements, which we attribute to the former more likely representing a combination of extrinsic and intrinsic piezoelectric contributions. To the best of our knowledge, this value (161.5 pC/N) is the highest reported for any halobismuthate, representing a nearly 4-fold enhancement as compared to published literature,^59^ as shown in Table S1 and Figure 4h. Moreover, the piezoelectric performance of (TMIM)_3_Bi_2_I_9_ is comparable to that of both BaTiO_3_ (*d*_33_ = 191 pm/V)^5^ and other higher-toxicity hybrid materials, while also demonstrating more advantageous phase transition characteristics (Figure 4i), maintaining a polar, noncentrosymmetric structure up to its decomposition temperature.

Our work shows that strategic design of organic–inorganic intermolecular interactions can yield high-performance hybrid piezoelectric materials. In the case of [(CH_3_)_3_NCH_2_I]_3_Bi_2_I_9_, the introduction of halogen bonding induces not only asymmetric positioning of the molecular components, but also structural distortion within the inorganic framework, ultimately resulting in a low-symmetry structure optimized for enhanced noncentrosymmetry and polarization. As a result, [(CH_3_)_3_NCH_2_I]_3_Bi_2_I_9_ demonstrates a piezoresponse close to that of BaTiO_3_, the highest reported for a halobismuthate (Figure 4h,i). Moreover, we demonstrate that the breaking of organic–inorganic halogen bonds within the (TMIM)_3_Bi_2_I_9_ structure drives a polar-to-polar phase transition at a high critical temperature (138 °C), with simultaneous order–disorder and displacive symmetry breaking. These findings provide strong experimental and theoretical evidence for the tunability of structural distortion and symmetry via careful manipulation of intermolecular interactions, thus validating and expanding on previous theories proposed to induce symmetry breaking in hybrid metal halides. This work establishes both a guiding design framework and a promising material system for the development of low-toxicity, high-performing piezoelectrics, enabling the next generation of sustainable, self-powered technologies.

## Supplementary Material



## Notes

Experimental section and additional figures and data: materials and synthesis, X-ray diffraction, NMR spectroscopy, PFM measurements, thermal analysis, piezoelectric measurements, first-principles calculations, optical measurements, point charge calculations, comparison of reported piezoelectric materials, additional electrostatic potential visualizations, crystallographic tables, thermal ellipsoid plots (PDF)
