# Supplementary material for: Tailoring a Lead-Free Organic–Inorganic Halobismuthate for Large Piezoelectric Effect
Source: J Am Chem Soc. 2025 Nov 25;147(49):45366–76. doi: 10.1021/jacs.5c15484 (PMC12703726; doi:10.1021/jacs.5c15484)
Supplement: Supplementary file 1 [file ja5c15484_si_001.pdf]

Supporting information for

Tailoring a lead-free organic-inorganic halobismuthate for large piezoelectric effect

Esther Y.H. Hung,[1] Benjamin M. Gallant,[2,3] Robert Harniman,[4] Jakob Möbs,[1,5] Santanu Saha,[1,6] Khaled Kaja,[7] Charles Godfrey,[1] Shrestha Banerjee,[2] Nikolaos Famakidis,[8] Harish Bhaskaran,[8] Paolo Radaelli,[1] Marina R. Filip,[1] Nakita K. Noel,[1] Dominik J. Kubicki,[2] Harry C. Sansom,*[1,4] Henry J. Snaith*[1]*

Author Address

[1] Clarendon Laboratory, Department of Physics, University of Oxford, Oxford OX1 3PU, United Kingdom

[2] School of Chemistry, University of Birmingham, Edgbaston, Birmingham B15 2TT, United Kingdom

[3] Department of Solution-Processing of Hybrid Materials and Devices, Helmholtz-Zentrum Berlin für Materialien und Energie GmbH, Berlin 12489, Germany

[4] School of Chemistry, University of Bristol, Bristol BS8 1TS, United Kingdom

[5] Institute for Inorganic and Analytical Chemistry, Justus-Liebig-University Gießen, Gießen 35392, Germany

[6] Institut de Recherche sur les Céramiques (IRCER), UMR CNRS 7315-Université de Limoges, Limoges 87068, France

[7] Bruker Nano Surfaces and Metrology, Karlsruhe 76187, Germany

[8] Department of Materials, Oxford, University of Oxford OX1 3PH United Kingdom

Contents

Experimental Section 4

Materials 4

Single crystal synthesis of $(TMIM)_3Bi_2I_9$ 4

Mechanosynthesis of $(TMIM)_3Bi_2I_9$ 4

Deposition of $(TMIM)_3Bi_2I_9$ thin films 4

X-ray Diffraction (XRD) 5

Solid state Nuclear Magnetic Resonance (ssNMR) spectroscopy 6

Piezoresponse force microscopy (PFM) 6

Differential Scanning Calorimetry 7

Thermogravimetric analysis 7

Piezoelectric measurements 7

Density Functional Theory (DFT) 7

Optical measurements 8

Notes 9

Note S1: Simple point charge model calculations 9

Note S2: Additional detail on NMR measurements 11

Tables 12

Table S1: Reported materials and their piezoelectric coefficients (d_{ij}) and phase transition temperatures (T_c). 12

Table S2: Calculated polarization vectors using Berry phase theory. 13

Table S3: Distances between the TMIM+ cation and the inorganic dimer at 0 °C and 140 °C, respectively. 14

Table S4: Crystallographic data for $(TMIM)_3Bi_2I_9$ from 273 – 343 K. 15

Table S6: Crystallographic data for $(TMIM)_3Bi_2I_9$ at 413K. 17

Figures 18

Figure S1: Polarization contributions from the $(Bi_2I_9)^{3-}$ dimer 18

Figure S2: Electrostatic potential (ESP) map of $TMIM^+$. 19

Figure S3: Electrostatic potential (ESP) map of $(TMIM)_3Bi_2I_9$ visualising the shared electron density between the halogen-bonded $TMIM^+$ cations with iodide. 20

Figure S4: Tilting of $(Bi_2I_9)^{3-}$ dimer with temperature. 21

Figure S5: Visualisation of distances used between the $TMIM^+$ cations and the $(Bi_2I_9)^{3-}$ dimer for Table 3. 22

Figure S6: Residual electron density map of for $(TMIM)_3Bi_2I_9$ at 140 °C, set at 0.5 eVÅ^{-3}. 23

Figure S8: Fitted powder and thin film XRD patterns of $(TMIM)_3Bi_2I_9$. 25

Figure S9: Piezoresponse force microscopy (PFM) of $(TMIM)_3Bi_2I_9$ thin films, 40 x 40 µm image. 26

Figure S10: PFM on thin films measured under variable DC bias. 27

Figure S11: Berlincourtmeter measurements, including photo of peak value. 28
Figure S13: Band structures obtained using DFT-PBE 30
Figure S14: Thermal ellipsoid plot of $(TMIM)_3Bi_2I_9$ at 273K, ellipsoids at 50% probability. 31
Figure S15: Thermal ellipsoid plot of $(TMIM)_3Bi_2I_9$ at 293K, ellipsoids at 50% probability. 32
Figure S16: Thermal ellipsoid plot of $(TMIM)_3Bi_2I_9$ at 313K, ellipsoids at 50% probability. 33
Figure S17: Thermal ellipsoid plot of $(TMIM)_3Bi_2I_9$ at 323K, ellipsoids at 50% probability. 34
Figure S18: Thermal ellipsoid plot of $(TMIM)_3Bi_2I_9$ at 333K, ellipsoids at 50% probability. 35
Figure S19: Thermal ellipsoid plot of $(TMIM)_3Bi_2I_9$ at 343K, ellipsoids at 50% probability. 36
Figure S20: Thermal ellipsoid plot of $(TMIM)_3Bi_2I_9$ at 353K, ellipsoids at 50% probability. 37
Figure S21: Thermal ellipsoid plot of $(TMIM)_3Bi_2I_9$ at 363K, ellipsoids at 50% probability. 38
Figure S22: Thermal ellipsoid plot of $(TMIM)_3Bi_2I_9$ at 373K, ellipsoids at 50% probability. 39
Figure S23: Thermal ellipsoid plot of $(TMIM)_3Bi_2I_9$ at 383K, ellipsoids at 50% probability. 40
Figure S24: Thermal ellipsoid plot of $(TMIM)_3Bi_2I_9$ at 393K, ellipsoids at 50% probability. 41
Figure S25: Thermal ellipsoid plot of $(TMIM)_3Bi_2I_9$ at 403K, ellipsoids at 50% probability. 42
Figure S26: Thermal ellipsoid plot of $(TMIM)_3Bi_2I_9$ at 413K, ellipsoids at 50% probability................ 43
Figure S27: Additional NMR measurements... 44
Bibliography 45

Experimental Section

Materials

Bismuth iodide (BiI_3, 99.999%) was purchased from Alpha Aesar Puratronic and (iodomethyl)-trimethylammonium iodide ((TMIM)I, 98+%) from Thermo Scientific Chemicals. Both were stored in a N_2-filled glovebox prior to use. Dimethyl sulfoxide (DMSO, 99.9%) and isopropanol alcohol (IPA, purity) and hydroiodic acid (HI, 57 wt% in H_2O, distilled, stabilized, 99.95%), diethyl ether (DEE, 99.0%) and Fomblin were purchased from Sigma Aldrich. Silver paint was purchased from RS pro.

Single crystal synthesis of $(TMIM)_3Bi_2I_9$

490.4 mg of (TMIM)I and 589.7 mg of BiI_3 were dissolved in 10 mL of HI and stirred vigorously at 90 ˚C for 4 h, then gradually cooled by 5 ˚C a day down to room temperature. The obtained crystals were separated, rinsed with DEE, and dried on a hotplate at 30 ºC for 15 minutes, resulting in dark red crystals with sizes ranging from 0.1 to 0.5 mm.

Mechanosynthesis of $(TMIM)_3Bi_2I_9$

490.4 mg of (TMIM)I and 589.7 mg of BiI_3 were weighed out into a Teflon grinding jar (14 mL) containing a zirconia ball bearing (10 mm), and ground in an electric ball mill (VWR Beater Mixer Mill) for 30 minutes at a vibration frequency of 15 Hz to produce a microcrystalline powder. This was then transferred to a glass vial and annealed for 30 minutes in an oven at 100 ˚C.

Deposition of $(TMIM)_3Bi_2I_9$ thin films

490.4 mg of (TMIM)I and 589.7 mg of BiI_3 were dissolved in 2.5 mL DMSO and the resulting solution was stirred vigorously for 2 h before being filtered through a PTFE membrane filter with a 0.45 μm pore size prior to thin film deposition. FTO-coated glass substrates were sequentially cleaned in ultrasonic baths for ten minutes each in the following solutions: deionized water with 4% v/v solution of Decon 90 cleaning detergent, deionized water, acetone and isopropanol (IPA). Substrates were then dried with a stream of compressed nitrogen gas and subjected to UV-ozone treatment for 20 minutes before deposition. 100 μL of the $(TMIM)_3Bi_2I_9$ precursor solution was then statically dispensed onto the substrates and spun at 4000 rpm (4000 rpm s^{-1}) for 30 seconds. 15 seconds into this protocol, 170 μL of IPA was dispensed onto the substrate to assist with the extraction of the solvent, DMSO, from the as-deposited thin film ("anti-solvent quench"). The films were then annealed at 100 °C for 30 minutes on a hotplate. All spin-coating and annealing was conducted inside a N_2-filled glovebox. The obtained films are approximately 100 nm thick.

X-ray Diffraction (XRD)

Single crystal X-ray diffraction (SCXRD) measurements.

Single crystals were dispersed in Fomblin on a microscope slide, and 100 µm-sized crystals were selected under a microscope and mounted on a 0.2 mm diameter MiTeGen microloop which was then transferred to the goniometer.

Variable temperature measurements from 0 – 150 °C were carried out at the I19 beamline at Diamond Light Source (session CY36669-1) using X-rays of energy 25 keV (λ = 0.48 Å) under a continuous N_2 stream, using φ-scans from –175 – 175°. The crystallographic data is shown in **Tables S4-6** and **Figures S14-26**, all taken on the same crystal. The transmission was maintained at a low level to minimise and prevent beam damage. For *in situ* electric-field measurements, single crystals were attached to a Kapton mount with copper wires as electrodes, using araldite epoxy. Silver paint was applied at each side of the crystal to ensure electrical contact. The Kapton mount was then attached to the adapted goniometer as seen in **Figure 3a**. The crystallographic *c* axis was identified by comparison to crystal morphology simulation using the Bravais Friedel Donnay Harker (BFDH) method in Mercury. [1] The observed voltage-dependent behaviour under negative bias rules out bias-induced thermal expansion, since thermal effects would occur under both positive and negative bias due to equivalent power dissipation regardless of voltage polarity. Lab-based SCXRD measurements were taken on a Rigaku Oxford Diffraction Supernova diffractometer with Mo Kα (λ = 0.71073 Å) wavelength.

Synchrotron diffraction data were indexed, integrated and reduced using *xia2* for small molecules with additional DIALS commands[2] to input the unit cell and space group and a resolution cut-off of 0.7 Å. Lab-based diffraction data was indexed, integrated and reduced using Rigaku CrysAlisPro v171.43.90. Structures were solved using SHELXT and refined anisotropically (except where stated, below) by full-matrix least-squares on F^2 using SHELXL in OLEX2-1.3.[3–7] For diffraction data taken at temperatures above 60 °C (333K), $TMIM^+$ groups were modelled using Fragment DB,[3] using the 0 °C (273 K) model. SADI restraints were used on the N-C distances in the $TMIM^+$ cations to enable a stable refinement. DELU restraints were used on most of the anisotropically refined carbon and nitrogen positions. For temperatures 130 °C (403 K) and 140 °C (413 K), partial occupancies were refined for the $TMIM^+$ groups. Furthermore, the structure at 140 °C exhibits to significant disorder in the $TMIM^+$ cations, resulting in total refined occupancies below 100% as described in the main text. For all structures, all non-hydrogen and non-cationic atoms were refined anisotropically. For the data taken at 140 °C (413 K), the disorder of the cationic units as well as low reflection intensities made it necessary to refine carbon positions of $TMIM^+$ isotropically. Hydrogen atoms were assigned to idealised rotating CH_3 geometric positions and idealised non-rotating CH_3 geometric positions, where the latter was used to enable stable refinement. For temperatures 130 °C (403 K) and 140 °C (413 K), hydrogen atoms were either partially or not assigned due to the disorder in the structure.

Powder XRD.

Powder and thin film XRD data were obtained using a Panalytical Empyrean X-ray diffractometer with a Cu $K\alpha_1$ wavelength (1.54060 Å) using a generator voltage and current of 40 kV and 40 mA, respectively. Bragg-Brentano geometry was used, using a fixed divergence of 0.5°. Data was then analysed using Rietveld refinement and Pawley fitting in Topas Academic.[8]

Solid state Nuclear Magnetic Resonance (ssNMR) spectroscopy

All solid-state nuclear magnetic resonance (NMR) spectroscopy was carried out using a Bruker Avance Neo 20.0 T spectrometer on mechanosynthesized $(TMIM)_3Bi_2I_9$ packed into 4 mm zirconia rotors. The temperature of the NMR rotors was varied from -40 to 150 °C and calibrated using the variation in ^{79}Br shift of KBr.[9] ^{1}H and ^{13}C NMR spectra were recorded under magic-angle spinning (MAS) with ^{1}H decoupling (20.0 T, 10 kHz), using a Hahn echo pulse sequence. All rotors were spun using dry nitrogen gas. Radiofrequency pulses of 76 kHz (^{1}H) and 57 kHz (^{13}C) were used. The recycle delay for ^{1}H and ^{13}C measurements was varied as a function of temperature to maintain a value of approximately 1.3 times the measured T_1. ^{1}H and ^{13}C spectra were referenced to solid adamantane (1.91 and 38.48 ppm, respectively). ^{209}Bi spectra (20.0 T) were acquired on static (non-spinning) samples using an RF pulse of 100 kHz. The transverse relaxation time constant (T_2') was found to be very short for ^{209}Bi, precluding use of train echo acquisition (Carr-Purcell-Meiboom-Gill, CPMG). Therefore, a variable-offset cumulative-spectrum (VOCS) method was employed, whereby the frequency of the transmitter was varied systematically over a wide frequency range and a series of subspectra were recorded. These subspectra were added to produce the final ^{209}Bi spectra shown at each temperature. Typically, between 5 and 30 subspectra were recorded spanning a range of 0.5 to 3 MHz. A Hahn echo pulse sequence was employed (RF pulse, 124 kHz), with a short (25 μs) echo delay and a recycle delay of 5 ms. For ^{14}N spectra two different pulse sequences were employed, depending on the width of the ^{14}N spectral envelope observed. At low temperatures (broad spectral envelope; slow $TMIM^+$ tumbling regime) a static wideband uniform-rate smooth truncation quadrupolar Carr-Purcell Meiboom-Gill (WURST-QCPMG) approach was employed, based on a previously reported method.[10] At high temperature (narrow spectral envelope; fast $TMIM^+$ tumbling regime) ^{14}N spectra were record *via* a Hahn echo pulse sequence (RF pulse, 70 kHz) under 4 kHz MAS in order to differentiate spectral features corresponding to $TMIM^+$ in the halobismuthate phase and the nitrogen-containing thermal degradation product observed. A recycle delay of 150 ms was used for all ^{14}N spectra. ^{14}N spectra were referenced to NH_4Cl (0 ppm).

Piezoresponse force microscopy (PFM)

Piezoresponse Force Microscopy (PFM) measurements were conducted using a Dimension Icon AFM system with a Nanoscope 6 controller. A conductive probe was utilised of spring constant and tip radius 0.1 N/m and 25 nm respectively, and the detection laser spot was placed at the electrostatic blind spot in order to minimise electrostatic contributions to the measurement.[11]

With the tip grounded, a 4 V alternating bias signal (V_{ac}) was applied to the sample. This was driven at the contact resonance of the first eigenmode of the cantilever with the tip in contact with the film. In this way magnitude of sample surface displacement and directionality were measured concurrent with topography through the amplitude and phase response of the cantilever for sample imaging.

To measure hysteresis in the piezoresponse of the material, force feedback maintained a constant tip contact force, off-resonance, whilst a DC bias voltage was swept from -3 V to +3 V. Amplitude and Phase response in the cantilever were therefore acquired relative to applied bias. From the gradient of the acquired curves the d_{33} coefficient could therefore be calculated. Hysteresis measurements were taken in locations crossing domains which presented different responses during PFM imaging of the surface. PFM measurements were also conducted using a Multi-Mode VIII AFM system with Nanoscope V controller. The same procedures were followed to generate correlative images of film piezoresponse in ambient environment.

Images were processed using the plane levelling function in Gwyddion[12] in order to remove the background.

Differential Scanning Calorimetry

Differential scanning calorimetry was measured using a TA Instruments Q2000 DSC configured with a Refrigerated Cooling System (RCS) instrument, at a rate of 10 °C/minute.

Thermogravimetric analysis

Thermogravimetric analysis (TGA) was conducted on a mechanosynthesized powder of $(TMIM)_3Bi_2I_9$ using a TA Instruments Q500 Thermogravimetric Analyser equipped with TA Instruments Thermal Advantage software. This instrument allows the measurement of weight change as a function of temperature/time under a controlled (N_2 gas) environment. Data analysis was performed using TA Instruments Universal Analysis data analysis program. The sample was heated at a rate of 10 °C per minute under a N_2 gas flow rate of 100 mL per minute. The mass of the sample was recorded as a function of temperature during the experiment.

Piezoelectric measurements

Piezoelectric coefficient measurements were conducted using an APC International Ltd YE2730A Berlincourt meter, which was first calibrated using a PZT reference.

Density Functional Theory (DFT)

Electrostatic potential (ESP) maps for $TMIM^+$ and $(TMIM)_3Bi_2I_9$ were computed using DFT as implemented in the Amsterdam Density Functional (ADF) module of the Amsterdam Modelling Suite (AMS 2023.104).[13,14] Single-point energy calculations were carried out on fixed geometries obtained from the crystal structures. For $TMIM^+$, in the form of a singly charged cation, the r^2SCAN-3c composite method was used.[15] This method combines the r^2SCAN meta-GGA exchange-correlation functional with a tailor-made triple-ζ polarized basis set (mTZ2P), and includes a D4 dispersion correction, geometrical counterpoise (gCP) correction for basis set superposition error, and additional short-range basis set incompleteness corrections. No frozen core approximation was used. The numerical integration quality was set to “good”, and the default Becke fuzzy cell integration grid was used.

For $(TMIM)_3Bi_2I_9$, in the form of a neutral molecular cluster, the BP86 generalized-gradient approximation (GGA) functional was used in combination with the TZP basis set and no frozen core approximation. Numerical integration quality was set to “normal”, and the default Becke fuzzy cell integration grid was used. ESP maps were visualized using ADF’s built-in tools by coloring electron density isosurfaces according to the SCF-derived electrostatic (Coulomb) potential. This approach gives a qualitative picture of the spatial charge distribution.

First principles modelling of structures based on $(TMIM)_3Bi_2I_9$ and $[(CH_3)_4N]_3Bi_2I_9$ was carried out via plane-wave density functional theory (DFT), as implemented in the Quantum Expresso-6.5 package.[16,17] We use the generalised gradient approximation, within the Perdew-Burke-Ernzerhof parametrization (DFT-PBE).[18] All calculations use the scalar relativistic Optimized Norm Conserving Vanderbilt pseudopotentials,[19] as collected in the Pseudo-Dojo database.[20] We used a kinetic energy cutoff of 70 Ry with gamma-centered *k*-point mesh of 6 x 4 x 2 for all calculations. The calculated band structures are shown in **Figure S13**.

To calculate the polarization vector of different structures, we used the Berry phase implementation of the Quantum Espresso code.[21–24] On top of gamma-centered *k*-point grid of 6 x 4 x 2 for scf calculations, we used

8 x 4 x 2, 6 x 8 x 2 and 6 x 4 x 5 k-point grid for the calculations of polarization vectors along [100] (P_x), [010] (P_y) and [001] (P_z) directions, respectively.

Calculations were performed using the experimental crystal structure of $(TMIM)_3Bi_2I_9$, obtained at 293 K (orthorhombic space group $Pna2_1$). $TMIM^+$ cations in this model were replaced with by $[(CH_3)_4N]^+$ by replacing the iodine in the $[(CH_3)_3NCH_2I]^+$ ($TMIM^+$) cation with H and relaxing these H atoms only. $Cs_3Bi_2I_9$ was obtained by replacing N of the TMIM cation with Cs and removing rest of the organic atoms of $TMIM^+$. We found that there was little difference in the polarization by interchanging Cs^+ and $[(CH_3)_4N]^+$ (**Table 2**), so calculations were also performed on the composition $Cs_3Bi_2I_9$ in the space group $P3_1c$ by modifying $[(CH_3)_4N]_3Bi_2I_9$ shown in **Figure 1b** and swapping $[(CH_3)_4N]^+$ with Cs^+, in order to calculate its polarization.[25]

Optical measurements

The total transmittance and total reflectance of samples were measured in a Cary 5000 spectrophotometer using an internal diffuse reflectance accessory. Absorbance was calculated according to $A = -ln(T + R)$ and the absorption coefficient $\alpha = \frac{A}{d}$; where d is the thickness of the thin film.

Notes:

Note S1: Simple point charge model calculations

For a unit cell of volume V and atoms with point charge q_i, a simple point charge calculation for polarization can be made using the following equation:

$$\boldsymbol{P} = \frac{1}{V} \sum_{\substack{atoms \\ in\, unit\, cell}} q_i \boldsymbol{r_i}$$

Where $\boldsymbol{r_i}$ denotes atomic positions with respect to the centre of the unit cell. We assign a charge +3 for Bi, -1 for I, and +1 for N.
E.g.:

$$\boldsymbol{P}_{dimer\ 1} = \frac{1}{V}\left(3\sum_1^2 \boldsymbol{r}_{Bi} - \sum_1^9 \boldsymbol{r}_I\right)$$

And using

$$\frac{eV}{Å^2} = 1.602\ \frac{\mu C}{cm^2}$$

1. **$(TMIM)_3Bi_2I_9$, 0°C**

Inorganic contribution:

Units of $\mu C/cm^2$	P_x	P_y	P_z (polar axis)	$\lvert \boldsymbol{P} \rvert$
Dimer 1	-12.21396763	2.128326589	-1.72544624	12.5175055
Dimer 2	-5.00238945	8.025027429	15.4909185	18.1492017
Dimer 3	5.002397119	15.08803816	7.330733542	17.504643
Dimer 4	12.21396379	9.428206204	15.4909185	21.8641382
**Net polarization ($\mu C/cm^2$)**	**3.83469×10^{-6}**	**34.66959838**	**36.58712431**	**50.4043522**

Organic contribution:

**Net polarization** ($\mu C/cm^2$)	0	3.60822×10^{-16}	-3.198164075	3.19816407

**Total point charge polarization (**$\mu C/cm^2$**)**	3.83469×10^{-6}	34.66959838	33.38896024	48.1331873

2. **$(TMIM)_3Bi_2I_9$, 140°C**

Inorganic contribution:

Units of $\mu C/cm^2$	P_x	P_y	P_z (polar axis)	| ***P*** |
Dimer 1	0	6.61723	0.10411	6.61085
Dimer 2	6.44714	13.4651	-11.2478	18.61919
**Net polarization from dimers**	**6.44714**	**20.0823**	**-11.1436**	**23.8457**

The organic contribution to polarization from the $TMIM^+$ cations are not calculated for $(TMIM)_3Bi_2I_9$ at 140°C due to their disorder in the structure.

3. **$[(CH_3)_4N]_3Bi_2I_9$**

Inorganic contribution:

Units of $\mu C/cm^2$	P_x	P_y	P_z (polar axis)	| ***P*** |
Dimer 1	-5.97953	3.45158	-15.61933	17.0772305
Dimer 2	5.97955	-3.45298	12.64892	14.4108775
**Net polarization from dimers**	**0.00002**	**-0.00140**	**-2.970411999**	**2.97041233**

Organic contribution:

**Net polarization** ($\mu C/cm^2$)	0.00000	-1.72478209	3.248958533	3.678397

**Total point charge polarization** ($\mu C/cm^2$)	0.00002	-1.72618	3.248958533	1.7485117

Note S2: Additional detail on NMR measurements

^{1}H and ^{13}C spin-lattice relaxation rate time constant

T_1 relaxation in solids is driven by local fluctuations in magnetic field, typically induced in organic molecules by dynamic motion including methyl group rotations or whole molecule tumbling, or the presence of nearby quadrupolar nuclei such as ^{127}I. We find that the ^{1}H and ^{13}C T_1 minima corresponding to the iodomethyl group of $TMIM^+$ occur at 42 °C and 0 °C, respectively; unusually low values for solids. We also find that the absolute values of T_1 for the iodomethyl groups (<1.2 s for ^{13}C T_1 at all temperatures investigated) are markedly shorter than those of the methyl environments of $(TMIM)^+$ (>10 s for ^{13}C T_1). This pronounced site-dependent relaxation behaviour suggests that the presence of quadrupolar ^{127}I nuclei may dominate the unusually fast ^{13}C and ^{1}H relaxation and unusually low temperatures of ^{13}C and ^{1}H T_1 minima. Similarly enhanced rates of longitudinal relaxation have been observed previously in organohalides and attributed to scalar relaxation of the second kind between the quadrupolar halogen nuclei and adjacent $^{13}C/^{1}H$.[26,27] Indeed, scalar relaxation and MAS-induced heteronuclear polarization exchange are well-established relaxation mechanism for nuclei coupled to halogens.[28,29] Notably, the distribution of T_1 values surrounding these minima is described by a quadratic, unless structural changes cause new T_1 relaxation mechanisms to become active as temperature increases. Fitting of the T_1 curves shows that there are substantial deviations in T_1 away from expected distribution at temperatures above 125 °C. This behaviour is consistent with a change in either the local environment or dynamic motion of the organic cation at this temperature.

^{14}N NMR spectra

In $TMIM^+$ we expect the strongly electronegative iodomethyl substituent to generate a large electric field gradient (EFG) tensor across the ^{14}N nucleus, giving rise to a large quadrupole coupling constant, C_Q, and thus a very broad ^{14}N spectral envelope. At 20 °C, this is observed. However, as has been reported previously for both $[CH_3NH_3]^+$ and $[(NH_2)_2CH]^+$ in 3D halide perovskites, if cation dynamics reorient the EFG tensor with a correlation time shorter than $1/C_Q$, the asymmetry of the cation structure is effectively averaged. In this circumstance, the EFG at the ^{14}N nucleus is determined not by the cation symmetry but by the local environment of the cation within its host structure. The larger distances between the cation and its surroundings mean this change invariably results in a reduced EFG at the nucleus, and thus a reduced C_Q and a pronounced narrowing of the ^{14}N spectral lineshape. The tenfold reduction in lineshape observed between ^{14}N NMR spectra of $[TMIM]_3Bi_2I_9$ above and below the phase transition is therefore strong experimental evidence that this transformation is driven by the onset of isotropic organic cation tumbling as halogen bond interactions break at elevated temperature, leading to an order-disorder type phase transition.

^{209}Bi NMR spectra

Release of the halogen bond interactions between the organic cation and inorganic dimer at the phase transition not only allows for the onset of rapid cation reorientation but also leads to a displacement of the I^- and Bi^{3+} ions with respect to one another in the inorganic dimer (the dimer partially symmetrises as a result of release of the halogen bonds). This displacement is observed directly via SCXRD (**Figure 2b**) and is consistent with the step-change in ^{209}Bi lineshape observed at the phase transition (**Figure 2g**). Furthermore, the point charge model indicates that this displacement reduces the inorganic contribution to polarization by more than half (**Note S1**). This displacement, and its experimental verification, are crucial to understanding the substantial polarization in the low-temperature phase of $(TMIM)_3Bi_2I_9$, as halogen bonding-induced asymmetry in the dimer significantly contributes to the polarization of the inorganic component (**Figure 1d**). The displacement of I^- and Bi^{3+} ions with respect to one another caused by symmetrisation of the dimer during the phase transition leads to a change in the EFG across the quadrupolar ^{209}Bi nucleus. The static lineshape of quadrupolar ^{209}Bi ($I = 9/2$) NMR spectra are dominated by C_Q. Thus, Given the extremely large magnitude of the quadrupole moment of ^{209}Bi (-422 mb),[30] approximately 25-fold larger even than that of ^{14}N), even a very slight change in EFG across the nucleus is expected to produce resolvable changes in the static ^{209}Bi NMR spectrum.

Tables

Table S1: Reported materials and their piezoelectric coefficients (d_{ij}) and phase transition temperatures (T_c).

	Material	d_{ij} (pC/N) (ij = 33 unless stated otherwise)	T_c (°C)	Reference
Ceramic oxides	PZT	360	350	[31]
	$BaTiO_3$ (BTO)	191	120	[32]
	$KNbO_3$	80-160	400	[33]
	$NaNbO_3$	52.5	370	[34]
Organic compounds	PVDF	34	154	[35]
	DIPAB	11	153	[36]
Cd,Pb, Mn-containing hybrids	$(TMCM)MnCl_3$	185	133	[37]
	$(TMBM)MnBr_3$	112	142	[38]
	$(TMCM)CdBr_3$	139	73	[39]
	$(TMFM)_{0.26}(TMCM)_{0.74}CdCl_3$	1540	80-100	[40]
	$(BnNMe_2Me)CdBr_4$	6	162 (melt)	
	$(FMTMA)PbCl_2I$	20.6	242	[41]
	$(ATHP)_2PbBr_4$	76	230	[42]
	$(BTMA)_2CoBr_4$	d_{25}= 12.4	140	[43]
	$(MDABCO)NH_4I_3$	14	175	[44]
Halobismuthates	**$(TMIM)_3Bi_2I_9$**	**161.5**	**138**	**This work**
	$(R/S\text{-}1,2\text{-}DAP\cdot I)_4\cdot I_3\cdot BiI_6$	d_{22} = 35	132	[45]
	$(R/S\text{-}\alpha\text{-}PEA)_4Bi_2I_{10}$	32	197	[46]
	$(Me_3S)_3Bi_2Br_9$	18	-	[47]
	$(Me_3NH)_3Bi_2I_9$	8	55	[48]
	$(Me(Ph)_3P)_3Bi_2Br_9$	8	187	[49]
	$\{(sCH(MePh)(Me)NH_3)BiBr_5\}_n$	2.6	240	[50]

Table S2: Calculated polarization vectors using Berry phase theory. All calculations in group (i) were conducted based on or by modifying the crystal structure of $(TMIM)_3Bi_2I_9$ in space group *Pna*2_1 at a temperature of 293 K (**Figure 1a** in main text). The calculation for $Cs_3Bi_2I_9$ ($[(CH_3)_4N]_3Bi_2I_9$) in group (ii) was conducted by replacing the $[(CH_3)_4N]^+$ cation with Cs^+ in the crystal structure of $(CH_3)_4N]_3Bi_2I_9$ in the space group $P3_1c$ as reported by Feldmann et al. (2001).[51]

Composition		Space group	Polarization vector (μC/cm²)			
			P_x	P_y	P_z	$\frac{\lvert P\rvert}{\lvert P_{ref}\rvert}$
(i)	$(TMIM)_3Bi_2I_9$	*Pna*2_1	0.000	3.57	0.000	10.2
(i)	$[(CH_3)_4N]_3Bi_2I_9$	*Pna*2_1	0.000	2.31	0.000	6.6
(i)	$Cs_3Bi_2I_9$	*Pna*2_1	0.000	2.28	0.000	6.5
(ii)	$Cs_3Bi_2I_9$ /$[(CH_3)_4N]_3Bi_2I_9$	$P3_1c$	0.000	0.000	0.035	1.0

Table S3: Distances between the TMIM+ cation and the inorganic dimer at 0 °C and 140 °C, respectively. See Figure 2.

Distances (Å)	0 °C	140 °C
N1-Bi1	13.333 (12)	12.749 (14)
N2-Bi1	10.687 (15)	10.87 (3)
N3-Bi1	9.434 (12)	9.29 (5)
N1-Bi2	10.099 (12)	9.359 (14)
N2-Bi2	6.528 (15)	6.69 (3)
N3-Bi2	6.637 (13)	6.54 (5)

Table S4: Crystallographic data for $(TMIM)_3Bi_2I_9$ from 273 – 343 K.

Temperature /K	**273**	**293**	**313**	**323**	**333**	**343**
Empirical formula	$C_{12}H_{33}Bi_2I_{12}N_3$	$C_{12}H_{33}Bi_2I_{12}N_3$	$C_{12}H_{33}Bi_2I_{12}N_3$	$C_{12}H_{33}Bi_2I_{12}N_3$	$C_{12}H_{33}Bi_2I_{12}N_3$	$C_{12}H_{33}Bi_2I_{12}N_3$
Formula weight	2160.17	2160.17	2160.17	2160.17	2160.17	2160.17
Crystal system	orthorhombic	orthorhombic	orthorhombic	orthorhombic	orthorhombic	orthorhombic
Space group	$Pna2_1$	$Pna2_1$	$Pna2_1$	$Pna2_1$	$Pna2_1$	$Pna2_1$
a/Å	29.91660(16)	29.93090(17)	29.94670(19)	29.9506(2)	29.9544(2)	29.9545(3)
b/Å	9.81640(6)	9.83970(6)	9.86750(6)	9.88250(7)	9.89870(8)	9.91700(10)
c/Å	14.16770(7)	14.18660(8)	14.21300(8)	14.22780(10)	14.24460(11)	14.26390(14)
α, β, γ /°	90	90	90	90	90	90
Volume/Å^3	4160.68(4)	4178.11(4)	4199.93(4)	4211.24(5)	4223.66(6)	4237.22(7)
Z	4	4	4	4	4	4
ρ_{calc}g/cm^3	3.449	3.434	3.416	3.407	3.397	3.386
μ/mm^{-1}	6.451	6.424	6.391	6.374	6.355	6.335
F(000)	3712	3712	3712	3712	3712	3712
Crystal size/mm^3	0.13 × 0.09 × 0.03	0.13 × 0.09 × 0.03	0.13 × 0.09 × 0.03	0.13 × 0.09 × 0.03	0.13 × 0.09 × 0.03	0.13 × 0.09 × 0.03
Radiation/Å	Synchrotron (λ = 0.4859)	Synchrotron (λ = 0.4859)	Synchrotron (λ = 0.4859)	Synchrotron (λ = 0.4859)	Synchrotron (λ = 0.4859)	Synchrotron (λ = 0.4859)
2Θ range for data collection/°	3.724 to 40	3.408177 to 40.582585	3.360848 to 40.615298	3.718 to 40	3.39888 to 40.583241	3.342686 to 39.778859
Index ranges	$-42 \le h \le 42$, $-11 \le k \le 13$, $-19 \le l \le 19$	$-42 \le h \le 42$, $-11 \le k \le 13$, $-19 \le l \le 19$	$-42 \le h \le 42$, $-11 \le k \le 13$, $-20 \le l \le 20$	$-42 \le h \le 42$, $-11 \le k \le 13$, $-20 \le l \le 20$	$-42 \le h \le 42$, $-11 \le k \le 13$, $-20 \le l \le 20$	$-42 \le h \le 42$, $-11 \le k \le 13$, $-20 \le l \le 20$
Reflections collected	40487	38081	36063	80301	28900	24914
Independent reflections	11810 [R_{int} = 0.0730, R_{sigma} = 0.0363]	11866 [R_{int} = 0.0661, R_{sigma} = 0.0330]	11943 [R_{int} = 0.0646, R_{sigma} = 0.0320]	11980 [R_{int} = 0.0672, R_{sigma} = 0.0332]	12023 [R_{int} = 0.0737, R_{sigma} = 0.0362]	12044 [R_{int} = 0.0882, R_{sigma} = 0.0428]
Data/restraints/parameters	11810/11/270	11866/38/270	11943/38/270	11980/53/270	12023/99/267	12044/99/267
Goodness-of-fit on F^2	1.051	1.038	1.093	1.092	1.087	1.118
Final R indexes [I >=2σ (I)]	R_1 = 0.0302, wR_2 = 0.0724	R_1 = 0.0321, wR_2 = 0.0859	R_1 = 0.0363, wR_2 = 0.1031	R_1 = 0.0391, wR_2 = 0.1079	R_1 = 0.0438, wR_2 = 0.1284	R_1 = 0.0493, wR_2 = 0.1394
Final R indexes [all data]	R_1 = 0.0329, wR_2 = 0.0754	R_1 = 0.0355, wR_2 = 0.0904	R_1 = 0.0411, wR_2 = 0.1100	R_1 = 0.0453, wR_2 = 0.1165	R_1 = 0.0539, wR_2 = 0.1457	R_1 = 0.0656, wR_2 = 0.1636
Largest diff. peak/hole / e Å^{-3}	1.71/-1.21	1.69/-1.50	1.82/-1.83	2.12/-1.95	2.64/-2.04	2.31/-2.32
Flack parameter	-0.007(5)	-0.011(5)	-0.015(6)	-0.021(5)	-0.009(6)	-0.017(7)

Table S5: Crystallographic data for $(TMIM)_3Bi_2I_9$ from 353 – 403 K.

Temperature /K	**353**	**363**	**373**	**383**	**393**	**403**
Empirical formula	$C_{12}H_{33}Bi_2I_{12}N_3$	$C_{12}H_{33}Bi_2I_{12}N_3$	$C_{12}H_{33}Bi_2I_{12}N_3$	$C_{12}H_{33}Bi_2I_{12}N_3$	$C_{12}H_{33}Bi_2I_{12}N_3$	$C_{12}H_{33}Bi_2I_{12}N_3$
Formula weight	2160.17	2160.17	2160	2160	2160	2160
Crystal system	orthorhombic	orthorhombic	orthorhombic	orthorhombic	orthorhombic	orthorhombic
Space group	$Pna2_1$	$Pna2_1$	$Pna2_1$	$Pna2_1$	$Pna2_1$	$Pna2_1$
a/Å	29.9482(3)	29.9339(3)	29.9141(3)	29.8895(3)	29.8367(3)	29.7386(4)
b/Å	9.93700(11)	9.96020(9)	9.98170(10)	10.00470(10)	10.03340(11)	10.07620(19)
c/Å	14.28570(15)	14.31310(12)	14.33970(13)	14.36930(13)	14.41040(13)	14.47960(18)
α, β, γ /°	90	90	90	90	90	90
Volume/Å^3	4251.36(8)	4267.42(7)	4281.74(7)	4296.93(7)	4313.95(8)	4338.84(12)
Z	4	4	4	4	4	4
ρ_{calc}g/cm^3	3.375	3.362	3.351	3.339	3.326	3.307
μ/mm^{-1}	6.314	6.29	6.269	6.247	6.222	6.186
F(000)	3712	3712	3712	3712	3712	3712
Crystal size/mm^3	0.13 × 0.09 × 0.03	0.13 × 0.09 × 0.03	0.13 × 0.09 × 0.03	0.13 × 0.09 × 0.03	0.13 × 0.09 × 0.03	0.13 × 0.09 × 0.03
Radiation/Å	Synchrotron (λ = 0.4859)	Synchrotron (λ = 0.4859)	Synchrotron (λ = 0.4859)	Synchrotron (λ = 0.4859)	Synchrotron (λ = 0.4859)	Synchrotron (λ = 0.4859)
2Θ range for data collection/°	3.339327 to 39.130358	3.72 to 39.99	3.370284 to 39.409219	3.726 to 40	3.732 to 39.998	3.746 to 40
Index ranges	$-42 \le h \le 42$, $-11 \le k \le 13$, $-20 \le l \le 20$	$-42 \le h \le 42$, $-11 \le k \le 13$, $-20 \le l \le 20$	$-42 \le h \le 42$, $-11 \le k \le 13$, $-20 \le l \le 20$	$-42 \le h \le 42$, $-11 \le k \le 13$, $-20 \le l \le 20$	$-42 \le h \le 42$, $-13 \le k \le 11$, $-20 \le l \le 20$	$-41 \le h \le 41$, $-13 \le k \le 11$, $-20 \le l \le 20$
Reflections collected	24004	80806	21144	81609	81999	82684
Independent reflections	12072 [R_{int} = 0.0894, R_{sigma} = 0.0430]	12088 [R_{int} = 0.0745, R_{sigma} = 0.0377]	12119 [R_{int} = 0.0716, R_{sigma} = 0.0365]	12182 [R_{int} = 0.0688, R_{sigma} = 0.0368]	12218 [R_{int} = 0.0566, R_{sigma} = 0.0317]	12260 [R_{int} = 0.0584, R_{sigma} = 0.0356]
Data/restraints/parameters	12072/99/265	12088/89/267	12119/99/264	12182/99/264	12218/129/262	12260/144/285
Goodness-of-fit on F^2	1.058	1.027	0.987	0.938	0.889	0.796
Final R indexes [I>=2σ (I)]	R_1 = 0.0481, wR_2 = 0.1404	R_1 = 0.0511, wR_2 = 0.1496	R_1 = 0.0523, wR_2 = 0.1534	R_1 = 0.0546, wR_2 = 0.1632	R_1 = 0.0557, wR_2 = 0.1680	R_1 = 0.0572, wR_2 = 0.1682
Final R indexes [all data]	R_1 = 0.0635, wR_2 = 0.1590	R_1 = 0.0680, wR_2 = 0.1691	R_1 = 0.0722, wR_2 = 0.1729	R_1 = 0.0797, wR_2 = 0.1841	R_1 = 0.0805, wR_2 = 0.1866	R_1 = 0.1006, wR_2 = 0.1915
Largest diff. peak/hole / e Å^{-3}	1.81/-2.25	2.46/-2.46	2.07/-2.37	1.97/-2.33	1.56/-2.34	1.48/-2.32
Flack parameter		-0.003(7)	-0.018(7)	-0.010(7)	-0.019(6)	0.000(6)

Table S6: Crystallographic data for $(TMIM)_3Bi_2I_9$ at 413K.

Temperature /K	**413**
Empirical formula	$Bi_2C_{8.46}I_{11.12}N_{2.12}$
Formula weight	1959.63
Crystal system	orthorhombic
Space group	$Pmn2_1$
a/Å	10.1691(4)
b/Å	14.6803(4)
c/Å	14.6936(3)
α, β, γ /°	90
Volume/Å^3	2193.54(11)
Z	2
ρ_{calc}g/cm^3	2.967
μ/mm^{-1}	5.885
F(000)	1641
Crystal size/mm^3	0.13 × 0.09 × 0.03
Radiation/Å	Synchrotron (λ = 0.4859)
2Θ range for data collection/°	3.33 to 40.612
Index ranges	$-13 \leq h \leq 11$, $-20 \leq k \leq 20$, $-20 \leq l \leq 20$
Reflections collected	44465
Independent reflections	6768 [R_{int} = 0.0369, R_{sigma} = 0.0246]
Data/restraints/parameters	6768/49/133
Goodness-of-fit on F^2	0.788
Final R indexes [I>=2σ (I)]	R_1 = 0.0592, wR_2 = 0.1792
Final R indexes [all data]	R_1 = 0.1062, wR_2 = 0.2131
Largest diff. peak/hole / e Å^{-3}	1.28/-1.14
Flack parameter	0.003(6)

Figures

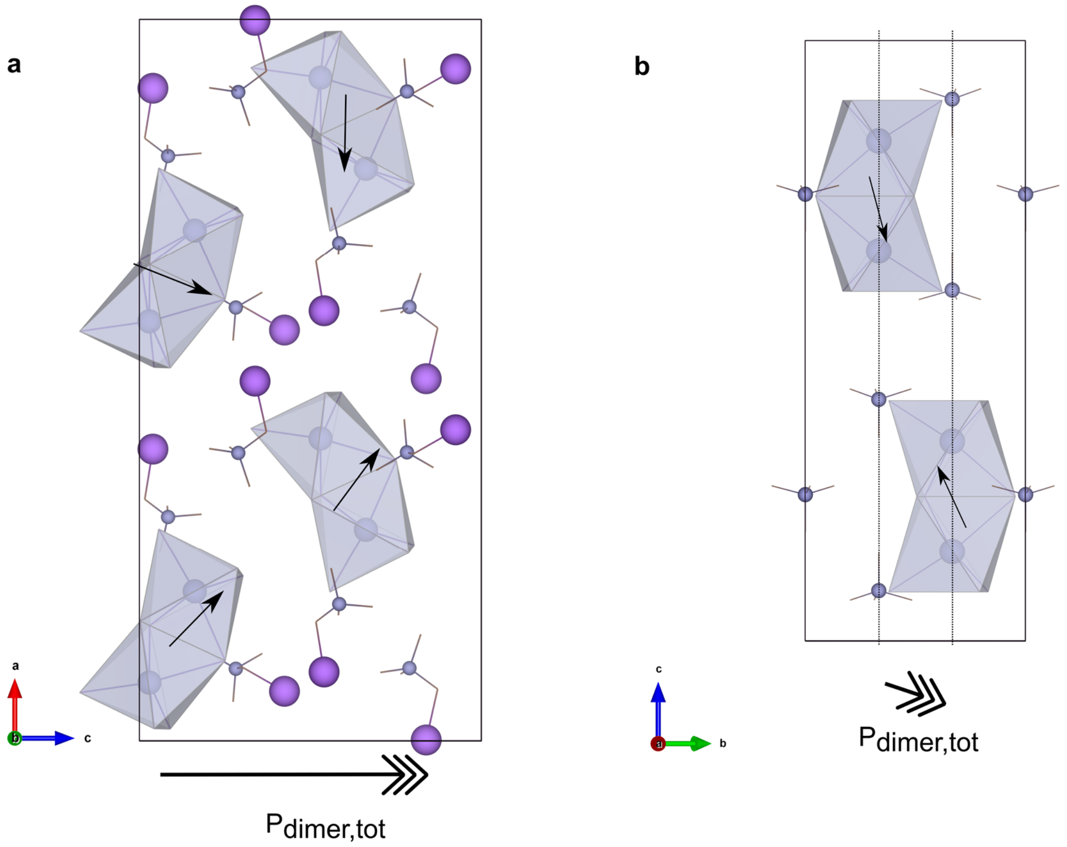

Figure S1: Polarization contributions from the $(Bi_2I_9)^{3-}$ dimer in **(a)** $(TMIM)_3Bi_2I_9$ and **(b)** $[(CH_3)_4N]_3Bi_2I_9$, respectively, at room temperature, using the point charge model.

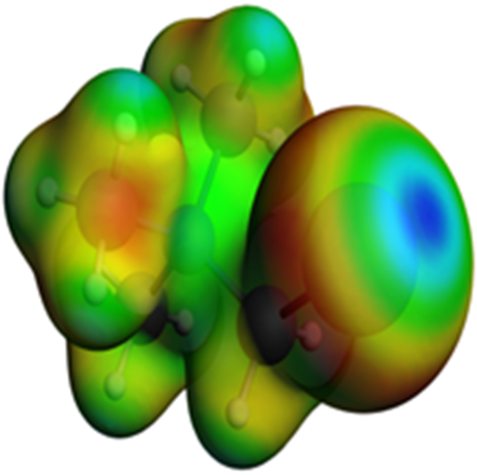

Figure S2: Electrostatic potential (ESP) map of $TMIM^+$. The color map represents the distribution of charge across the cation; red and blue correspond to regions of lesser and greater positive charge, respectively.

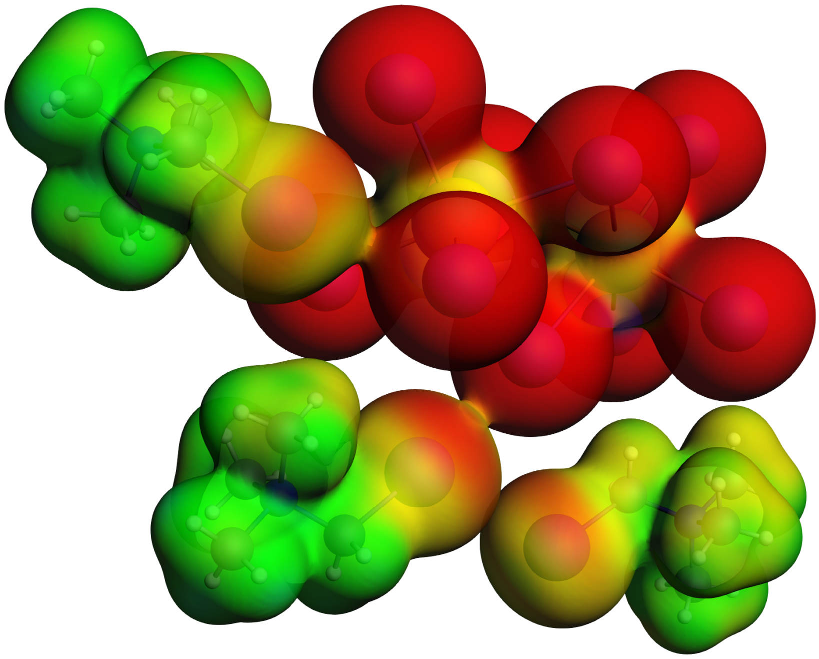

Figure S3: Electrostatic potential (ESP) map of $(TMIM)_3Bi_2I_9$ visualising the shared electron density between the halogen-bonded $TMIM^+$ cations with iodide.

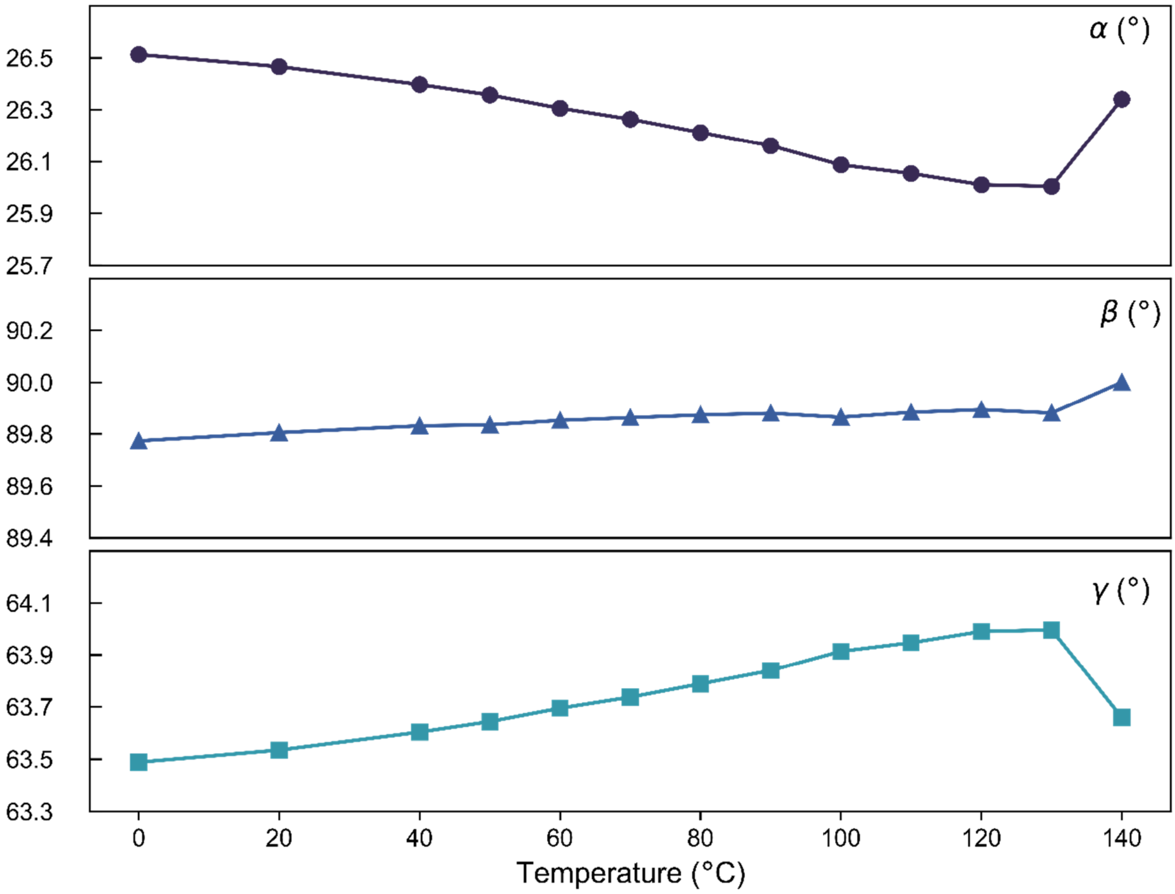

Figure S4: Tilting of $(Bi_2I_9)^{3-}$ dimer with temperature. α, β and γ refer to the angle with respect to the *a*, *b* and *c*-axis, respectively.

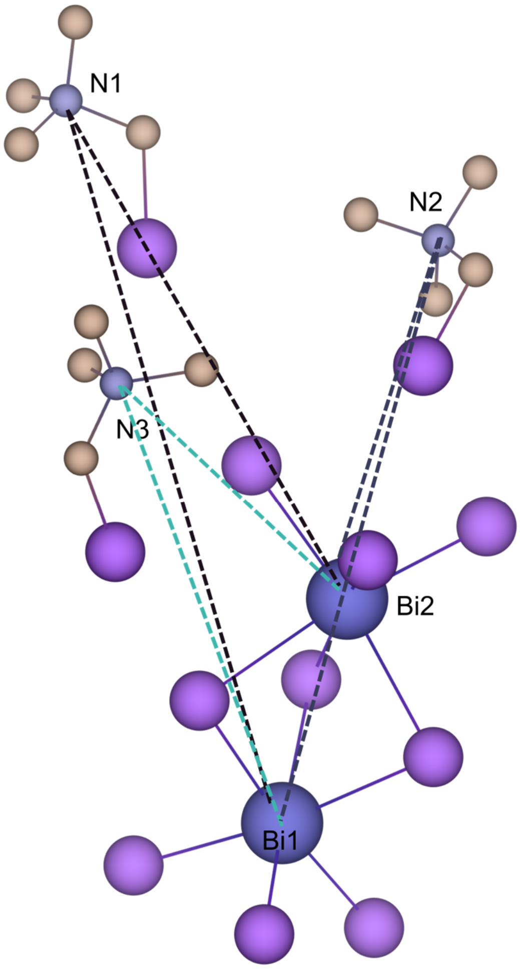

Figure S5: Visualisation of distances used between the TMIM$^+$ cations and the $(Bi_2I_9)^{3-}$ dimer for Table 3.

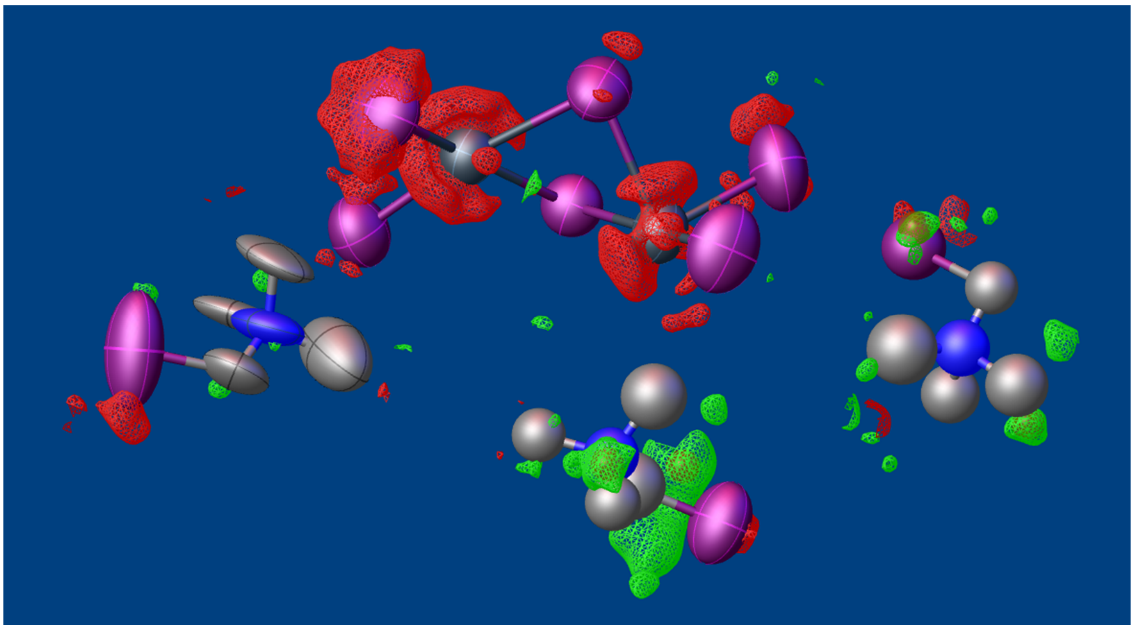

Figure S6: Residual electron density map of for $(TMIM)_3Bi_2I_9$ at 140 °C, set at 0.5 eÅ^{-3}.

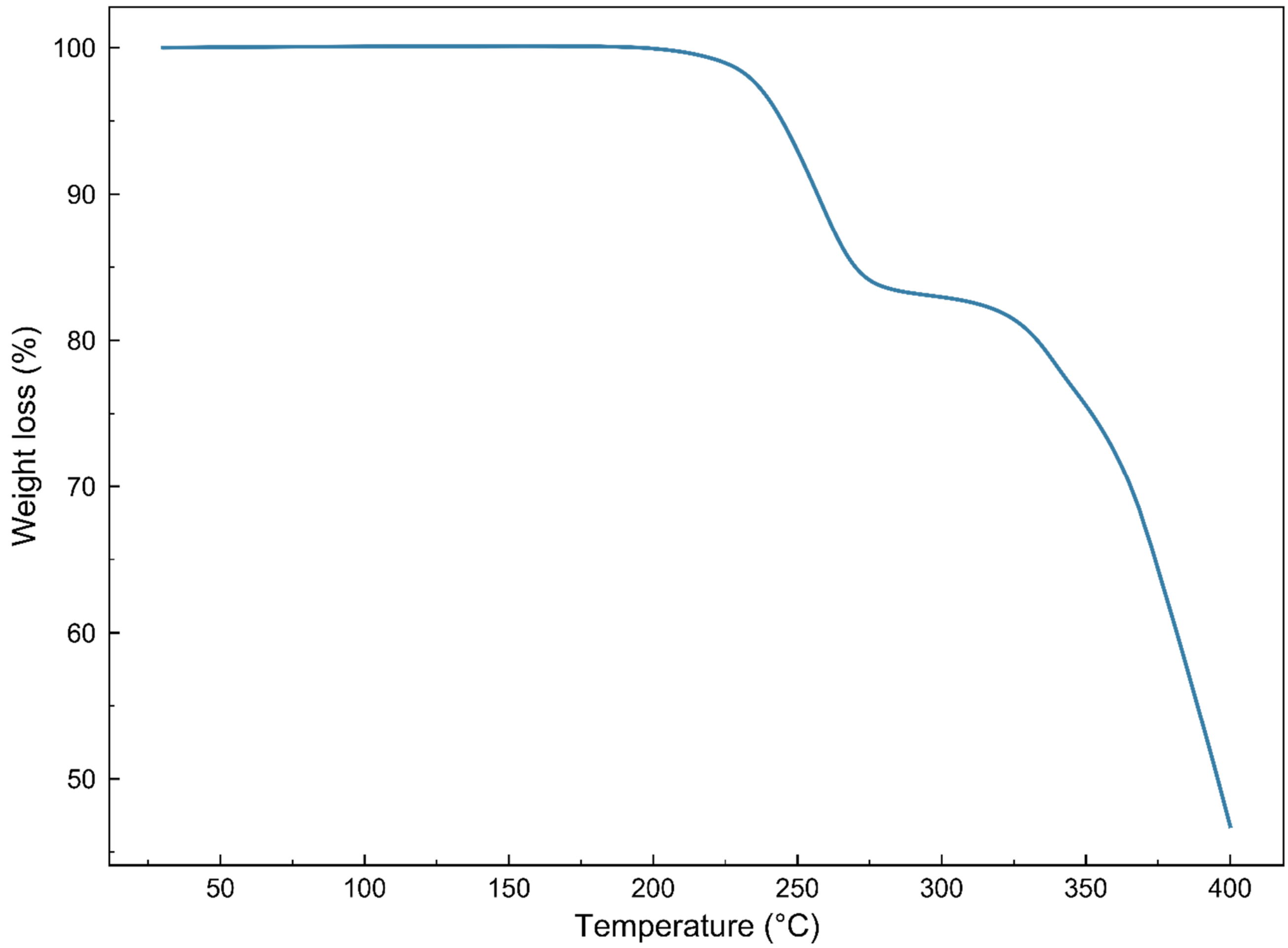

Figure S7: Thermogravimetric analysis (TGA) of $(TMIM)_3Bi_2I_9$

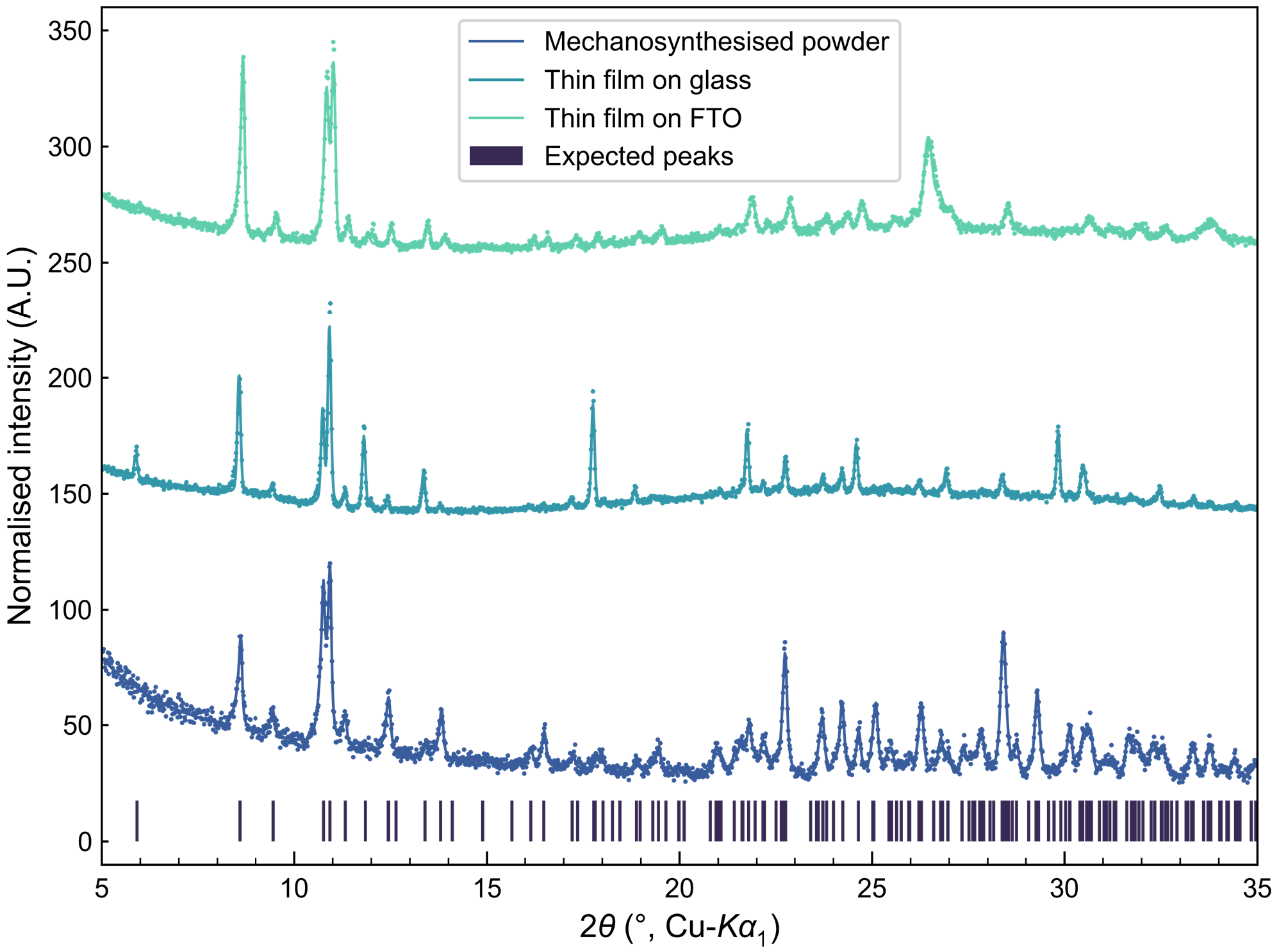

Figure S8: Fitted powder and thin film XRD patterns of $(TMIM)_3Bi_2I_9$.

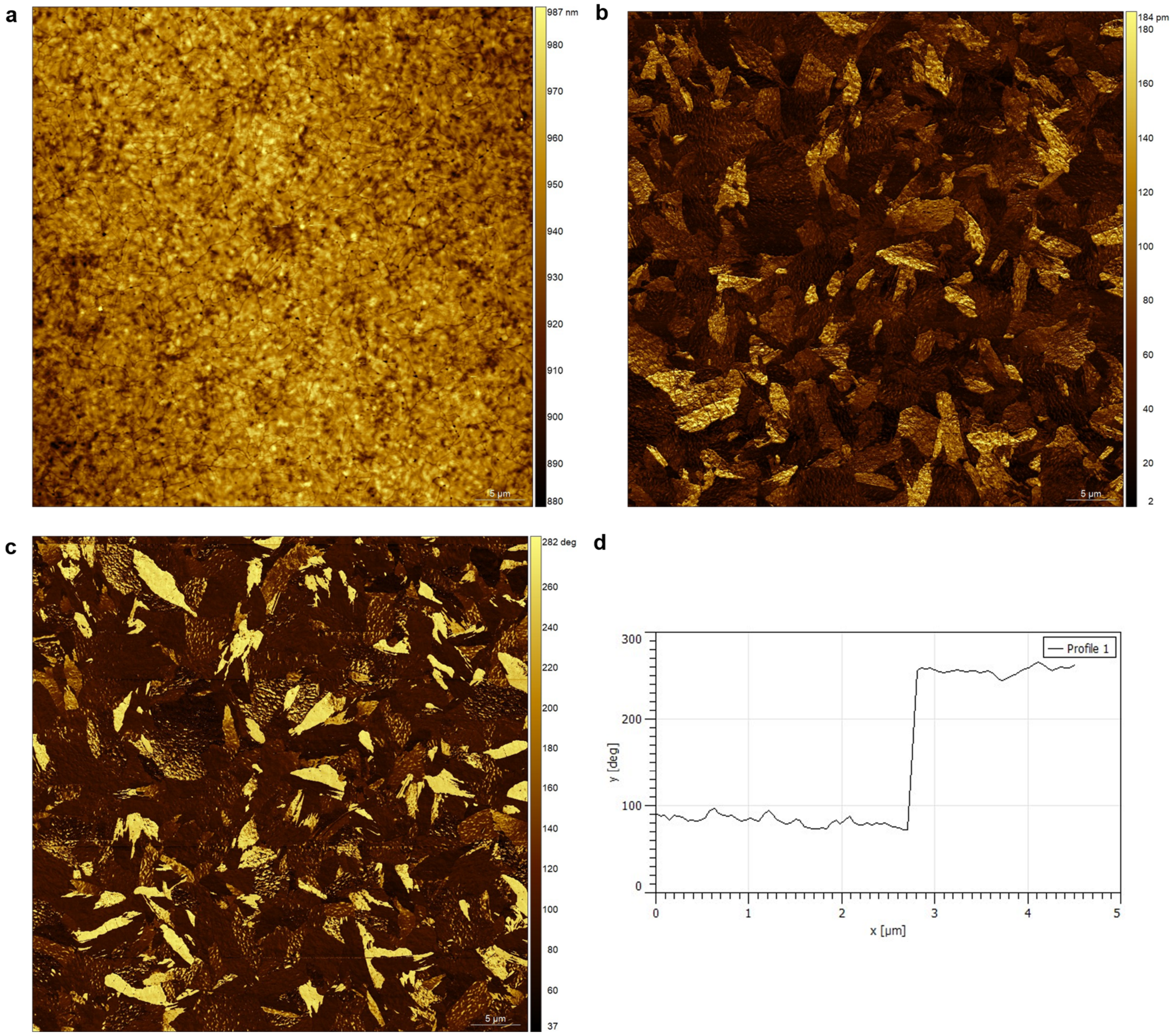

Figure S9: Piezoresponse force microscopy (PFM) of $(TMIM)_3Bi_2I_9$ thin films, 40 x 40 µm image. **(a)** Topography, **(b)** PFM amplitude and **(c)** PFM phase and **(d)** line profile indicating 180 ° domains

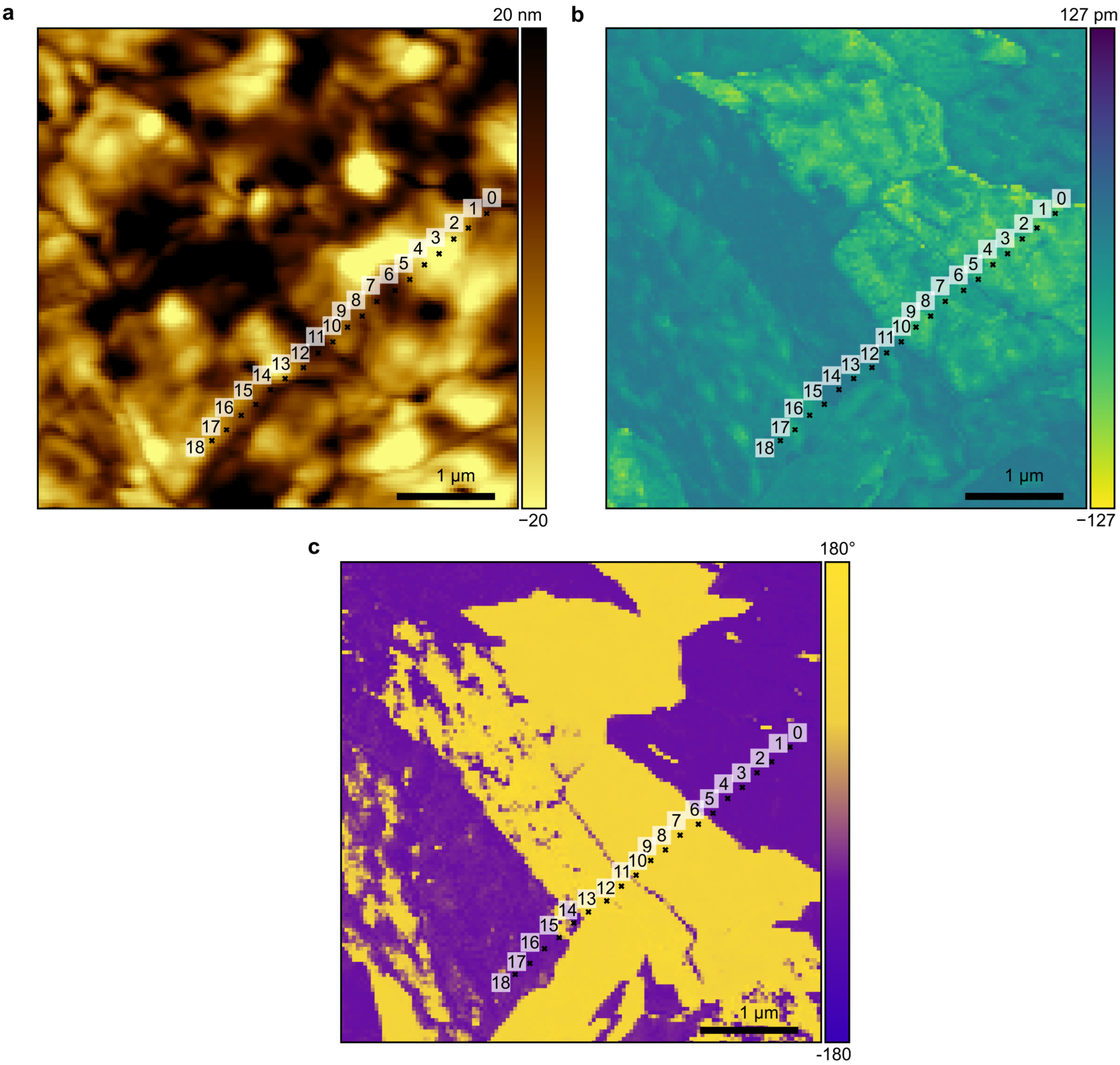

Figure S10: PFM on thin films measured under variable DC bias. **(a)** topography **(b)** amplitude **(c)** phase image used for obtaining amplitude- and phase-voltage loop measurements. Measurement points used are indicated with numbers.

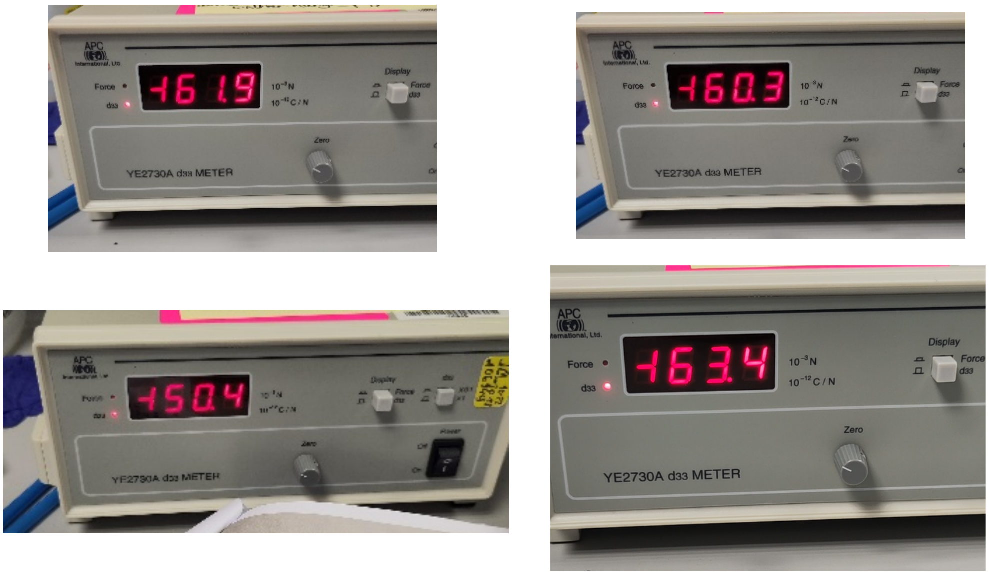

Figure S11: Berlincourtmeter measurements, including photo of peak value.

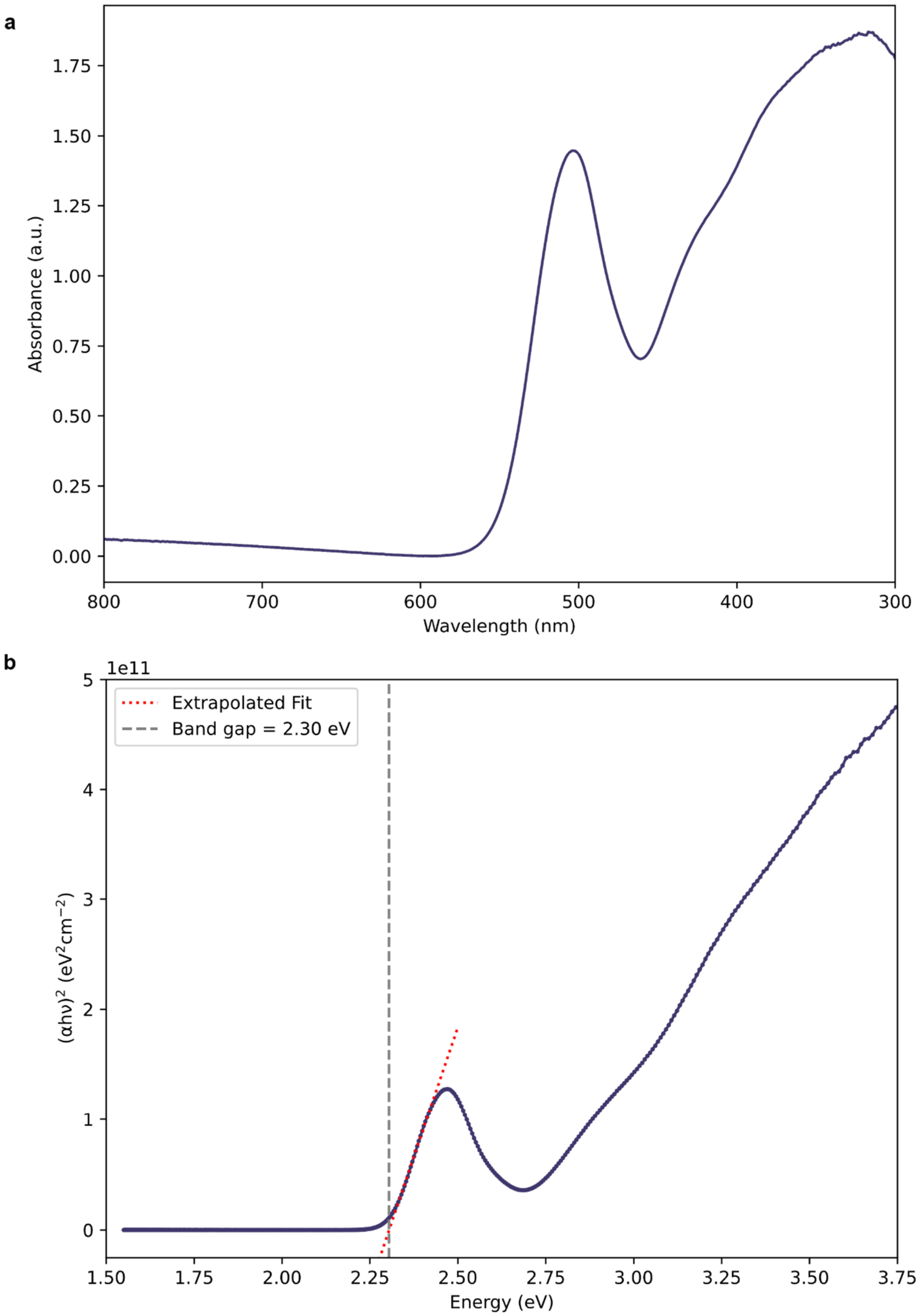

Figure S12: UV-vis absorption spectra of $(TMIM)_3Bi_2I_9$ thin films on glass. **(a)** Absorbance, taken as $Absorbance = -\ln(T + R)$; where T and R are the total transmittance and reflectance, respectively. **(b)** Tauc plot, assuming a direct band gap E_g, leads to optical $E_g = 2.30\ eV$.

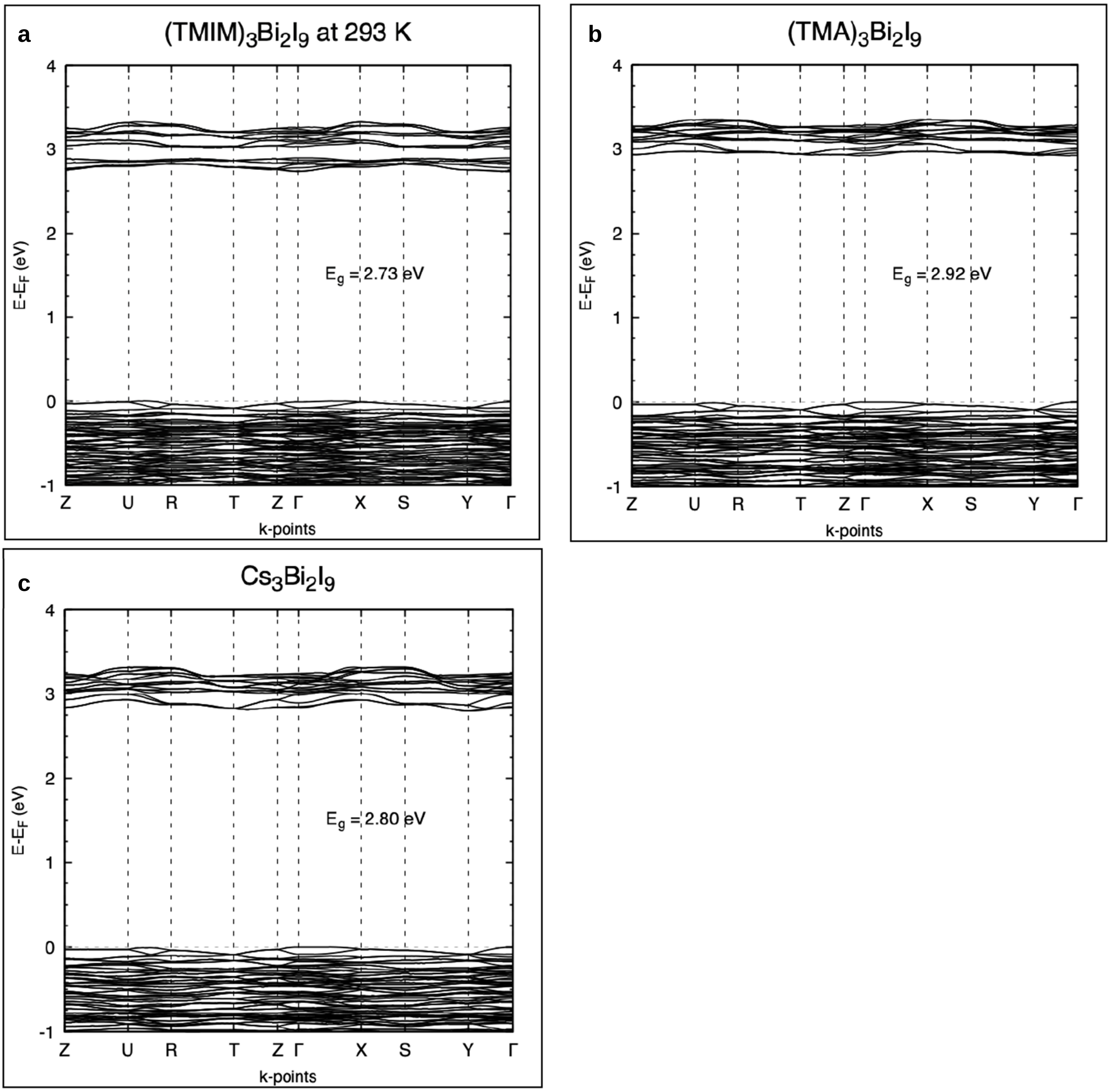

Figure S13: Band structures obtained using DFT-PBE calculations for **(a)** $(TMIM)_3Bi_2I_9$ **(b)** $[(CH_3)_4N]_3Bi_2I_9$ and **(c)** $Cs_3Bi_2I_9$, based on the original or modified crystal structure of $(TMIM)_3Bi_2I_9$ in space group $Pna2_1$. All calculations were performed using the experimental crystal structures measured at 293 K, as a starting point.

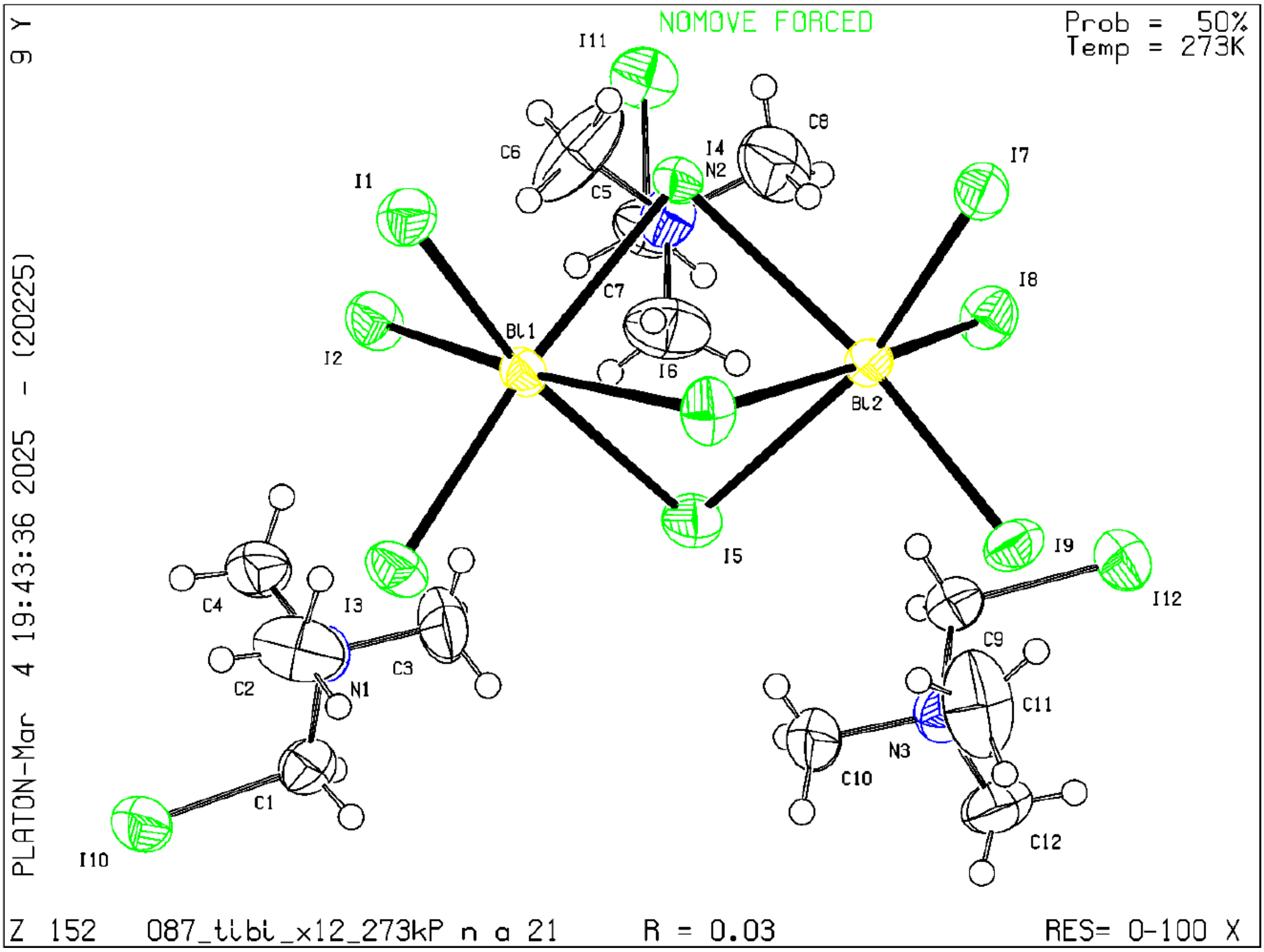

Figure S14: Thermal ellipsoid plot of $(TMIM)_3Bi_2I_9$ at 273K, ellipsoids at 50% probability.

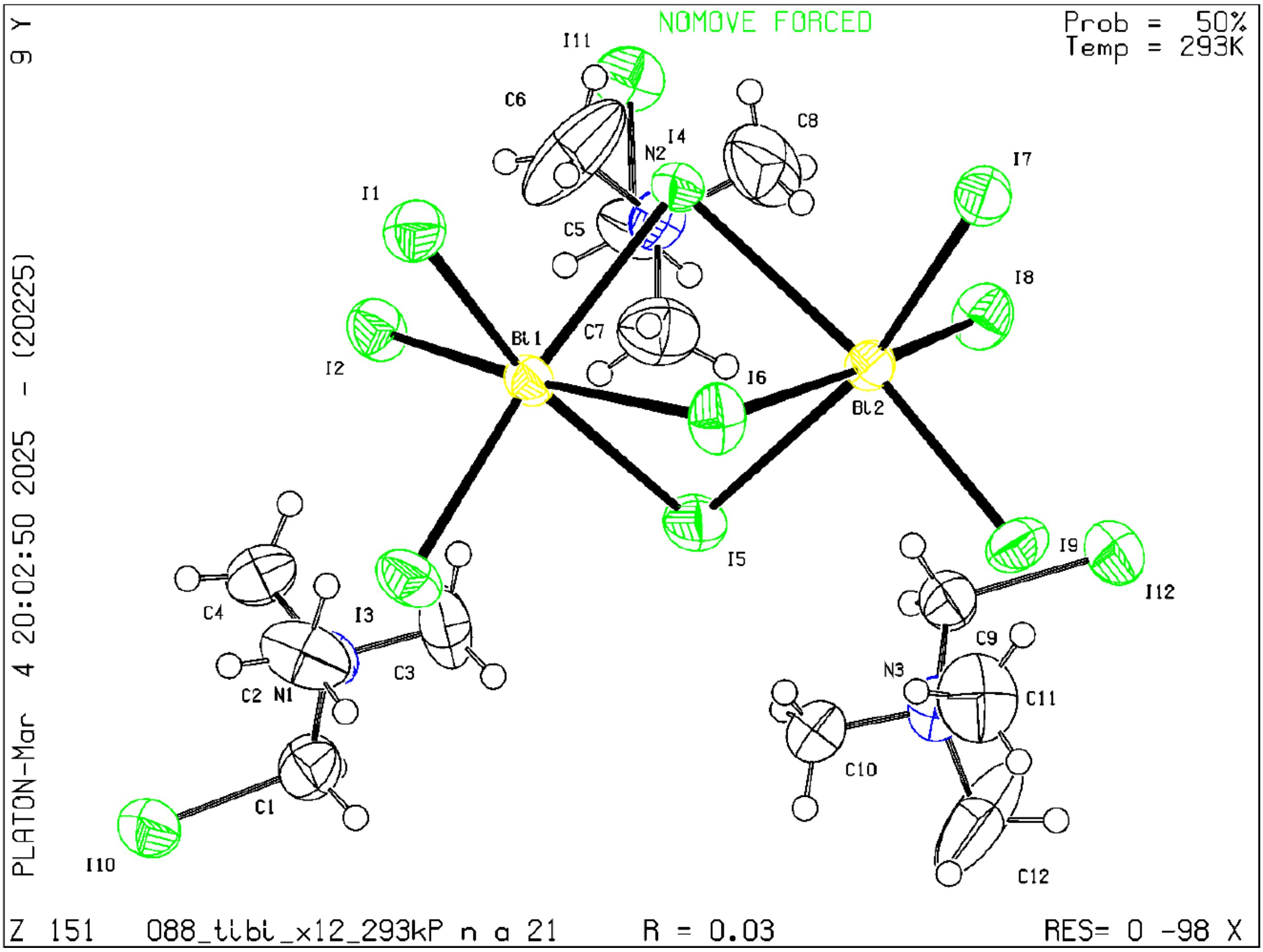

Figure S15: Thermal ellipsoid plot of $(TMIM)_3Bi_2I_9$ at 293K, ellipsoids at 50% probability.

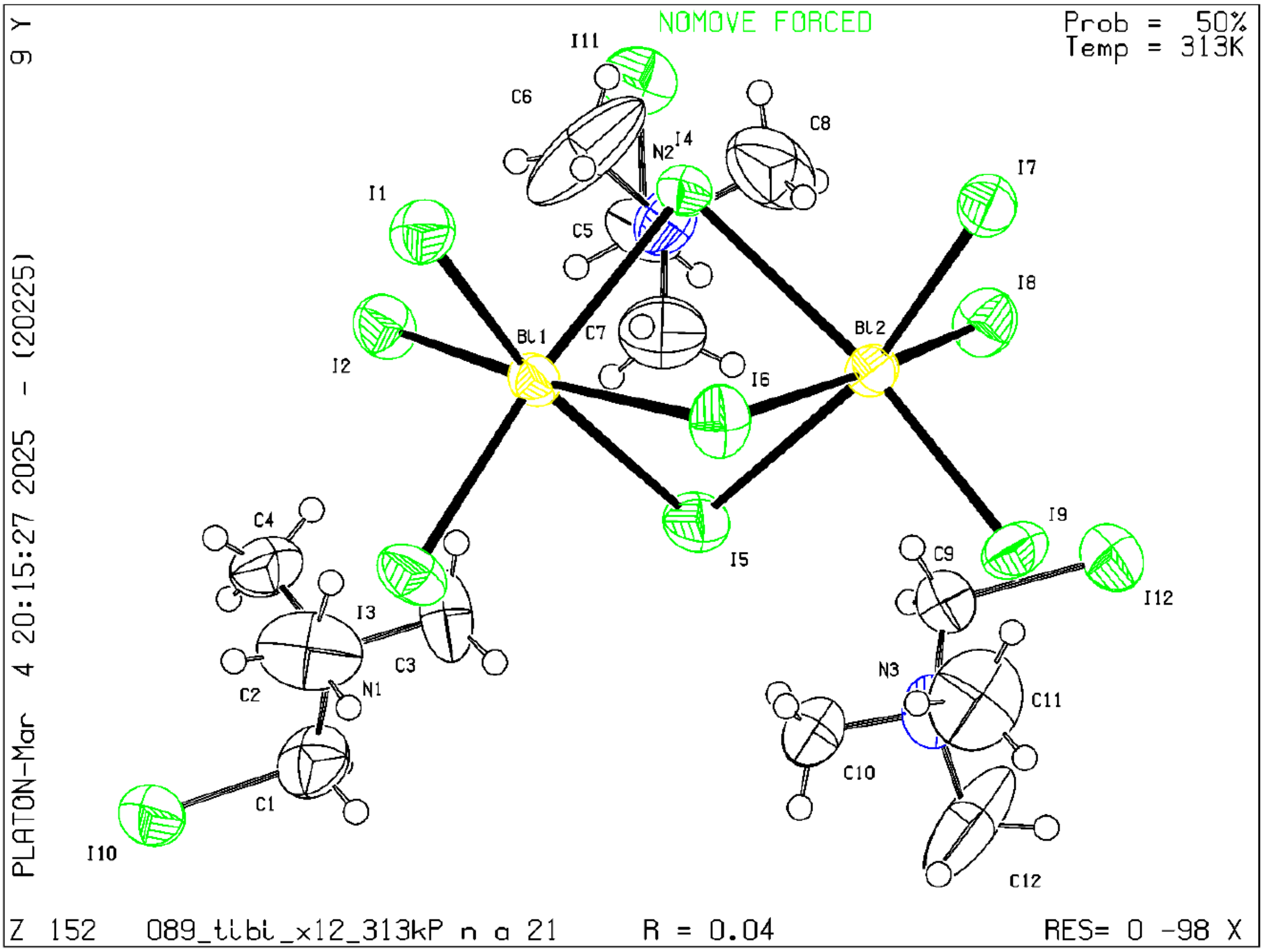

Figure S16: Thermal ellipsoid plot of $(TMIM)_3Bi_2I_9$ at 313K, ellipsoids at 50% probability.

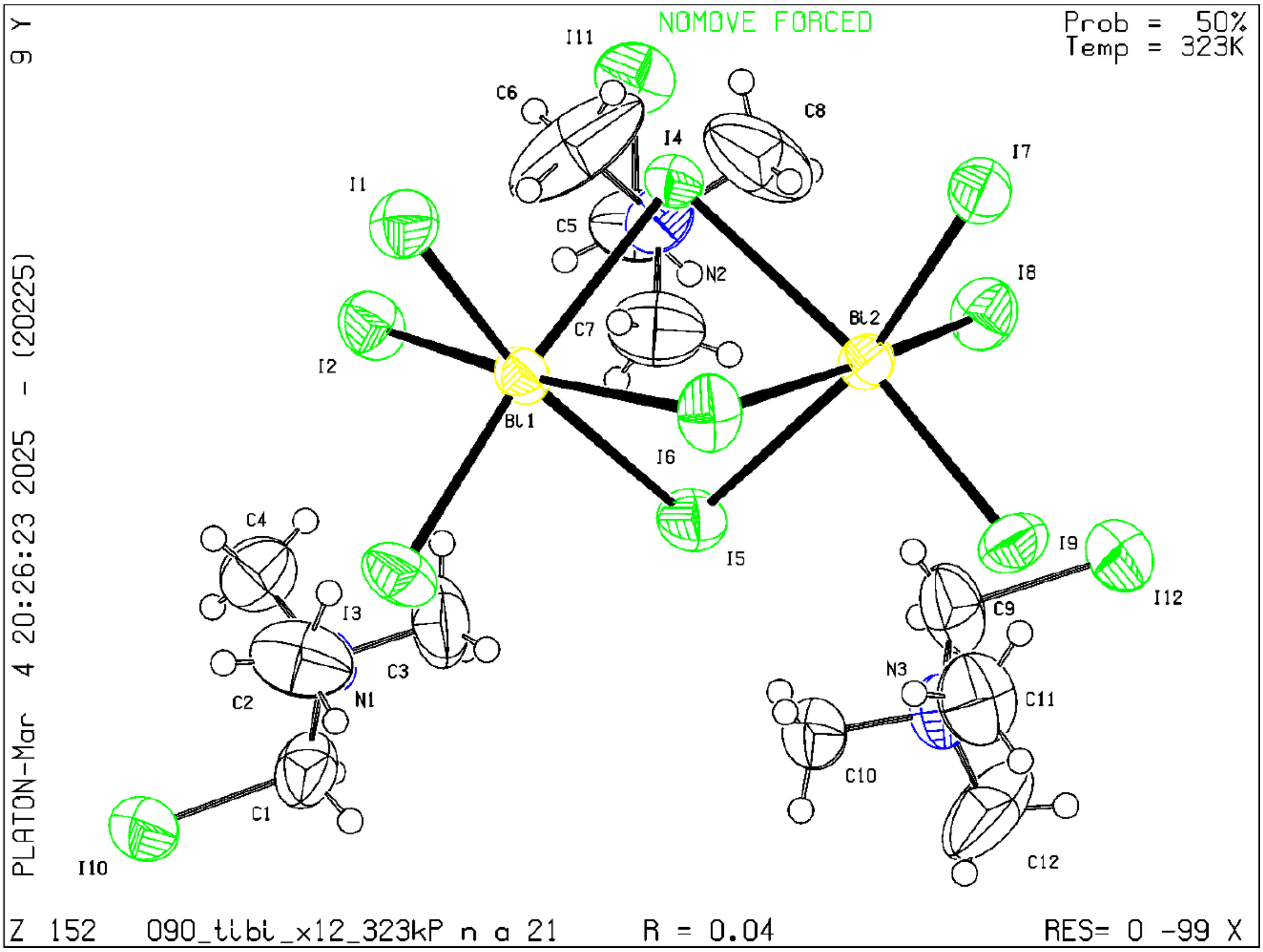

Figure S17: Thermal ellipsoid plot of $(TMIM)_3Bi_2I_9$ at 323K, ellipsoids at 50% probability.

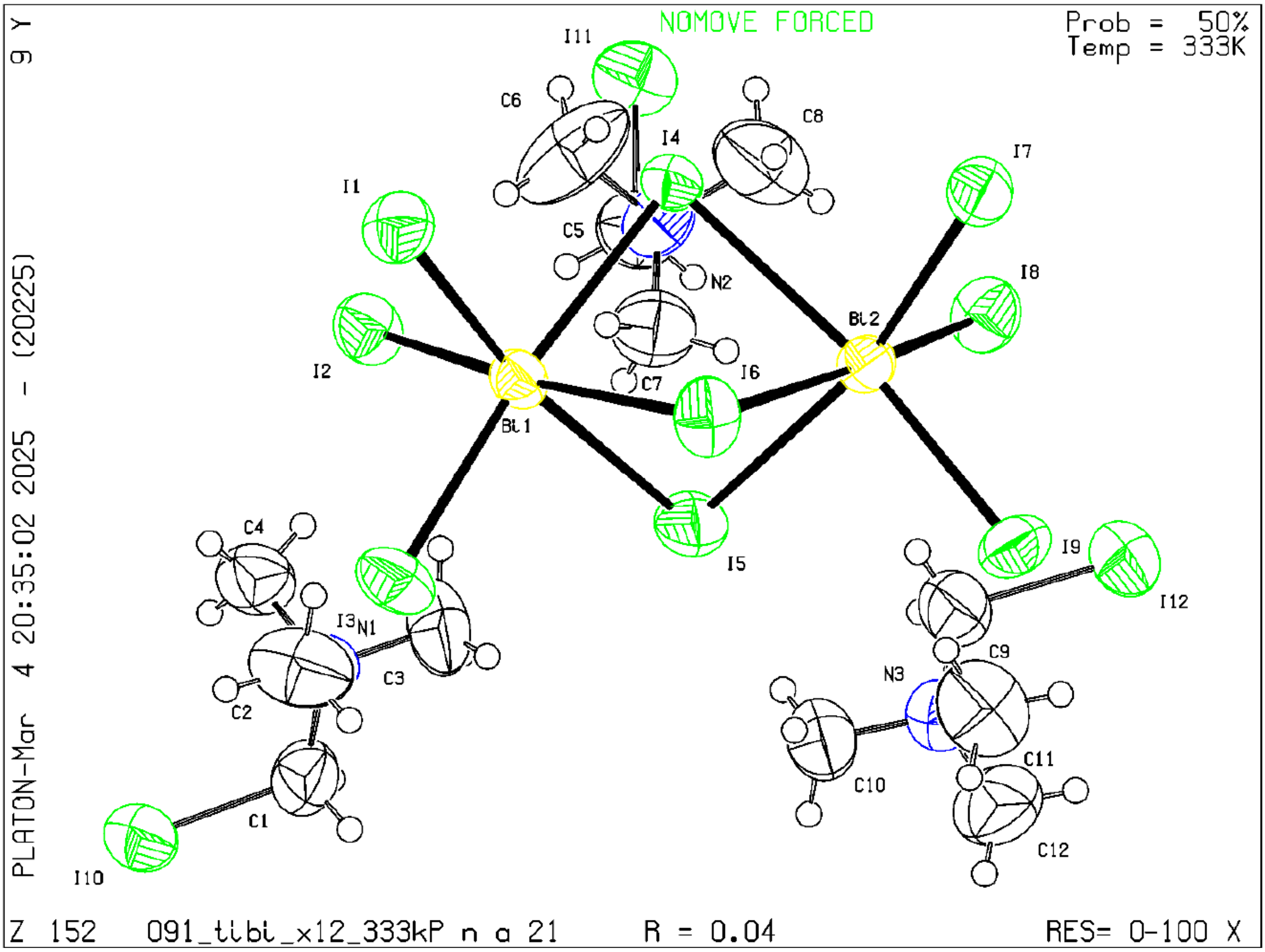

Figure S18: Thermal ellipsoid plot of $(TMIM)_3Bi_2I_9$ at 333K, ellipsoids at 50% probability.

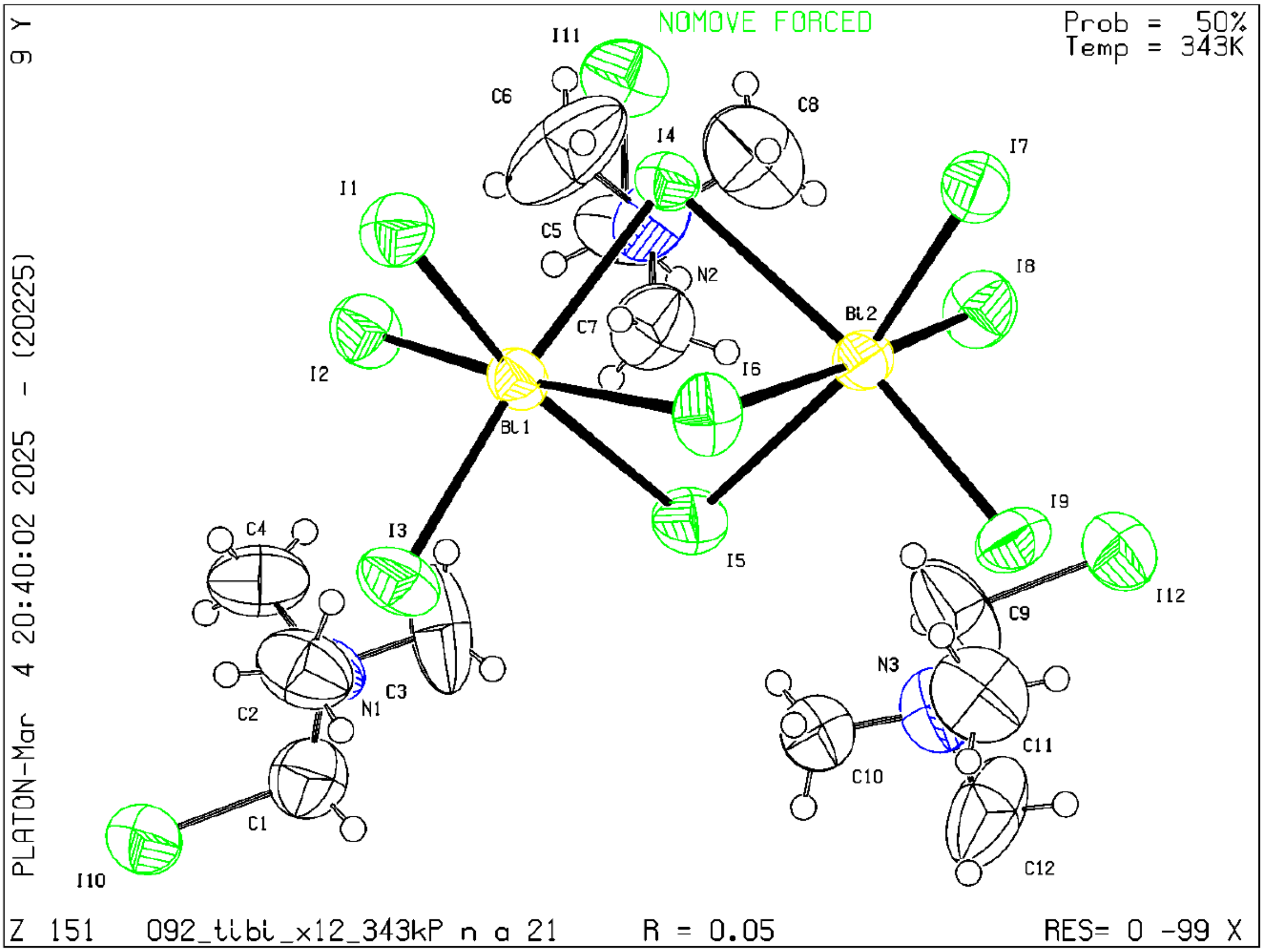

Figure S19: Thermal ellipsoid plot of $(TMIM)_3Bi_2I_9$ at 343K, ellipsoids at 50% probability.

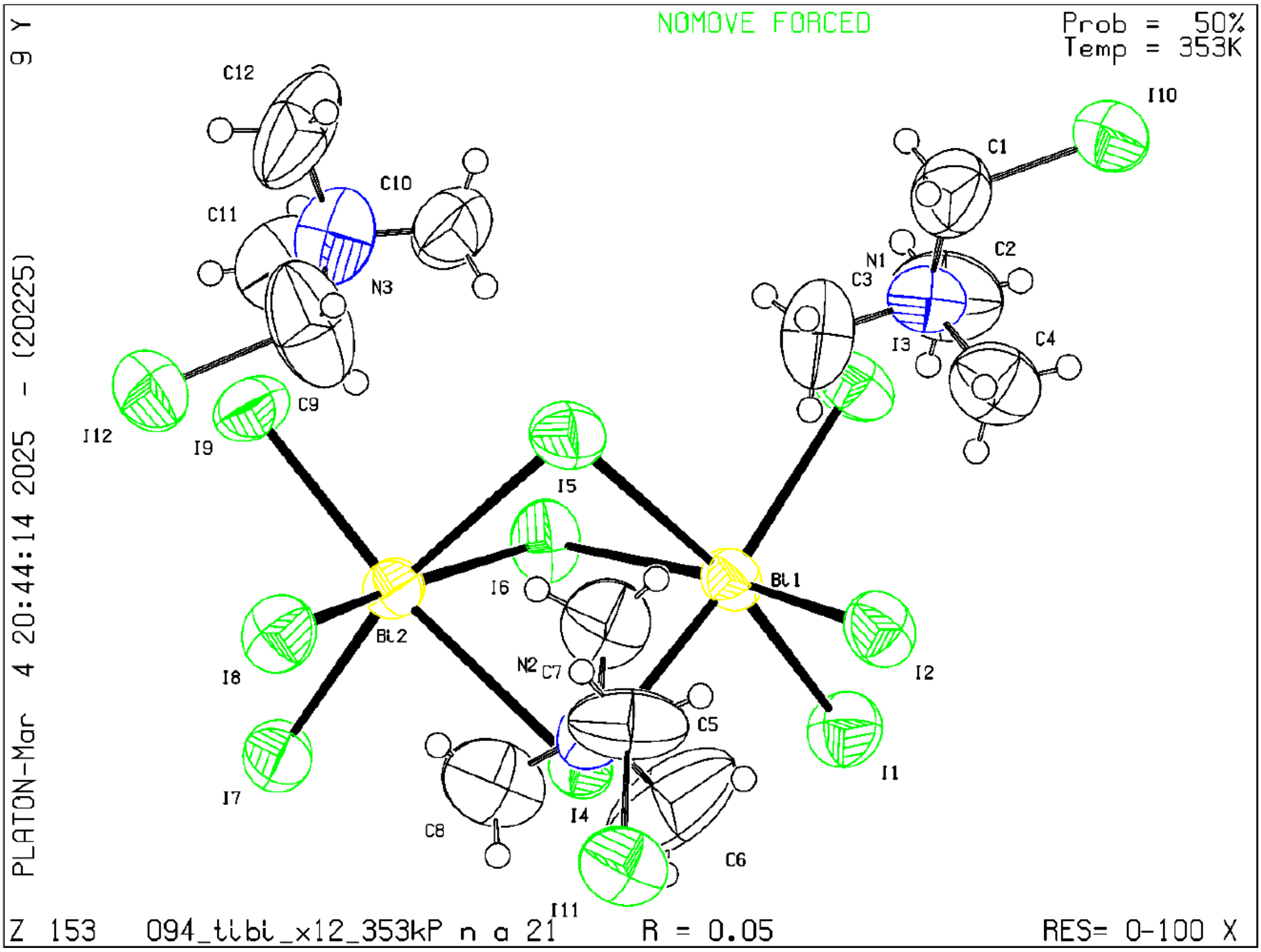

Figure S20: Thermal ellipsoid plot of $(TMIM)_3Bi_2I_9$ at 353K, ellipsoids at 50% probability.

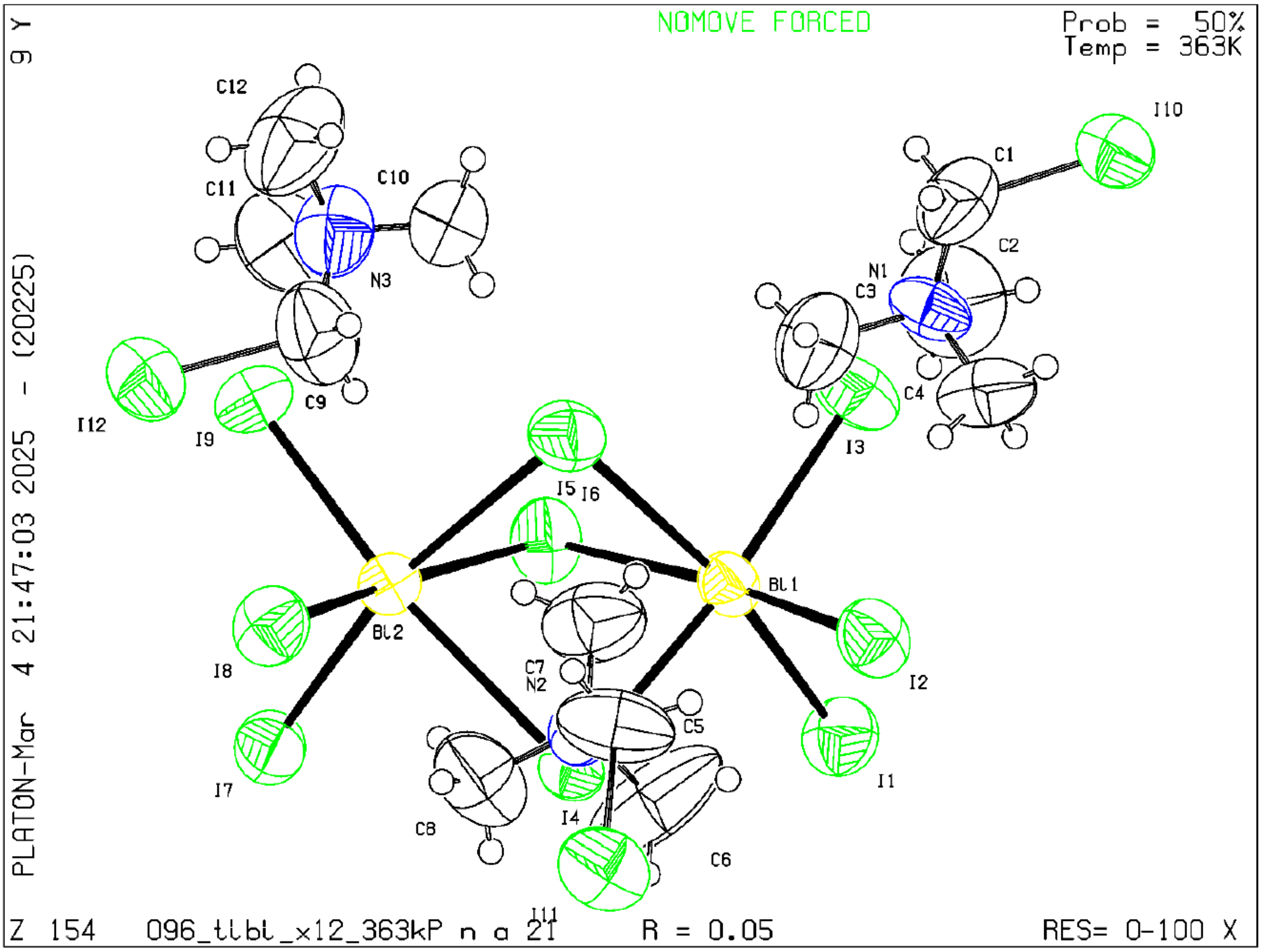

Figure S21: Thermal ellipsoid plot of $(TMIM)_3Bi_2I_9$ at 363K, ellipsoids at 50% probability.

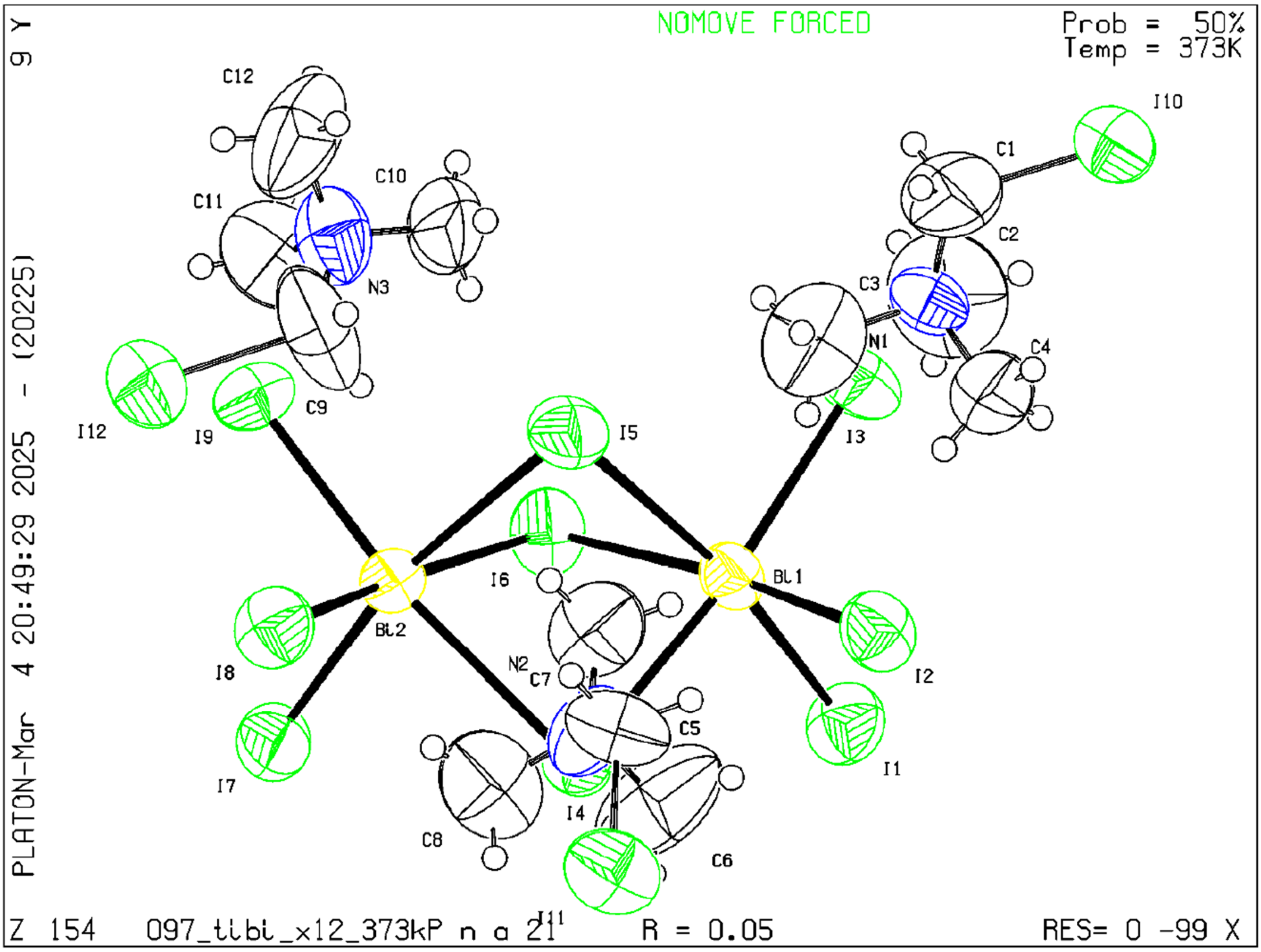

Figure S22: Thermal ellipsoid plot of $(TMIM)_3Bi_2I_9$ at 373K, ellipsoids at 50% probability.

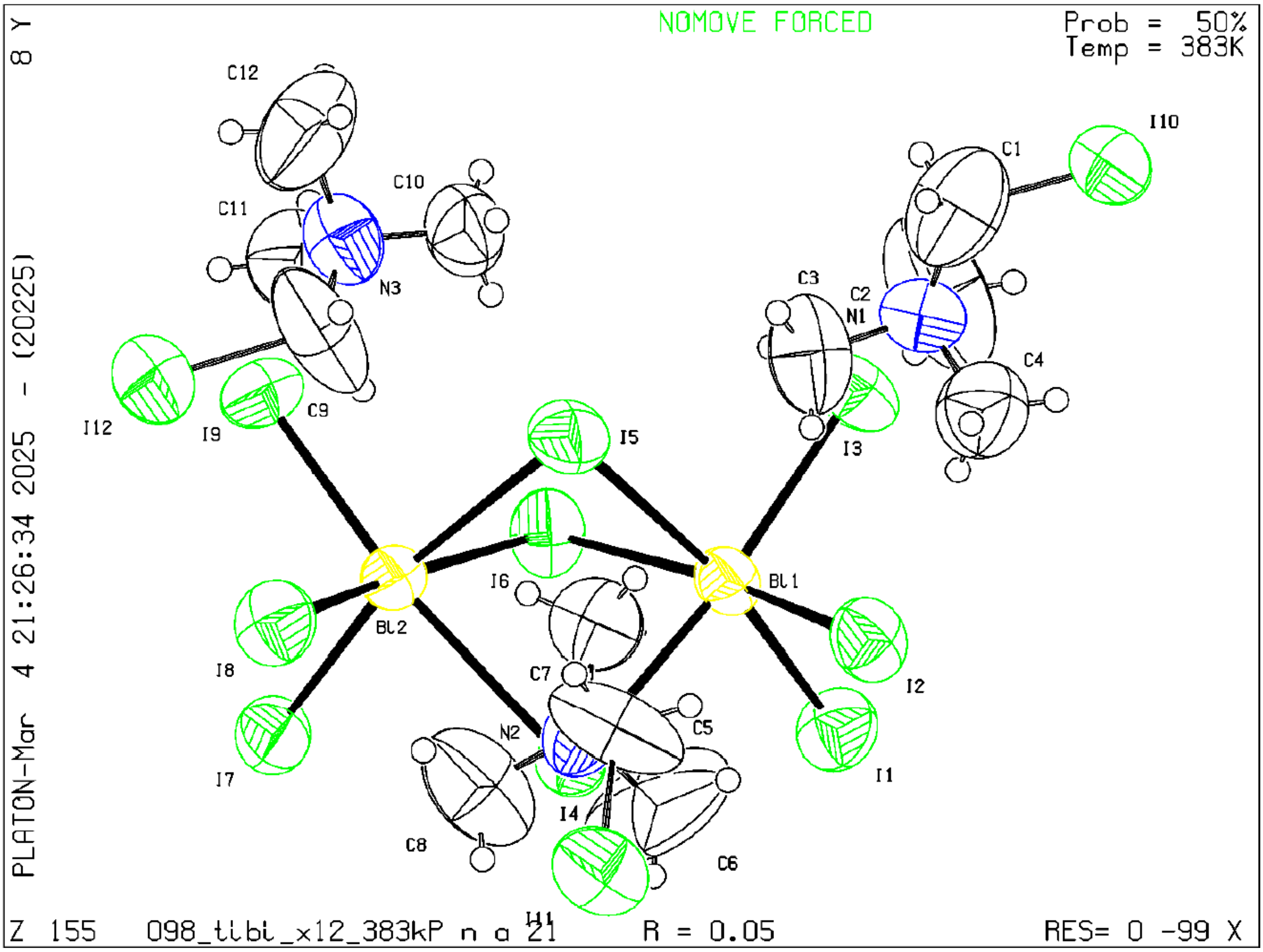

Figure S23: Thermal ellipsoid plot of $(TMIM)_3Bi_2I_9$ at 383K, ellipsoids at 50% probability.

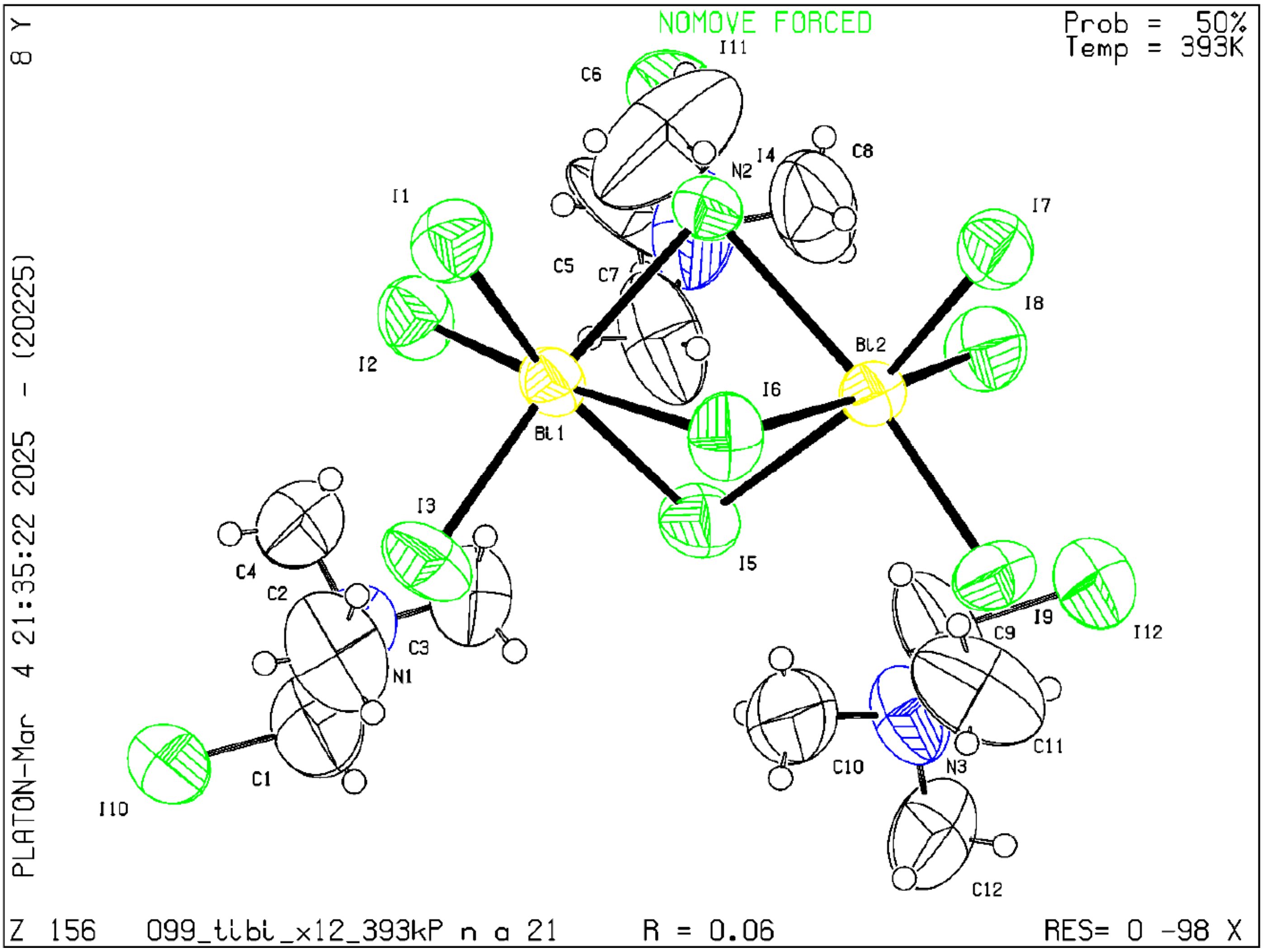

Figure S24: Thermal ellipsoid plot of $(TMIM)_3Bi_2I_9$ at 393K, ellipsoids at 50% probability.

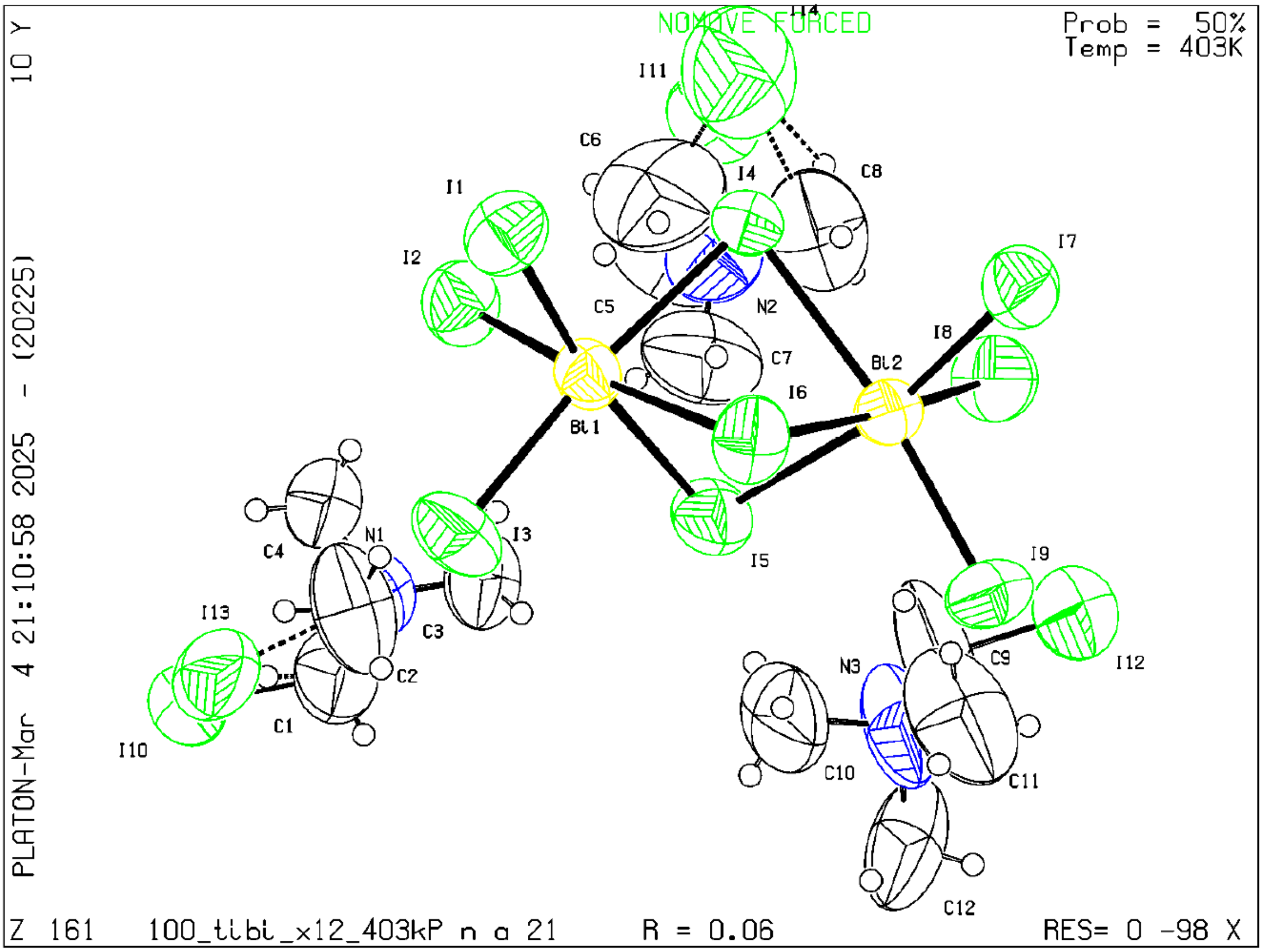

Figure S25: Thermal ellipsoid plot of $(TMIM)_3Bi_2I_9$ at 403K, ellipsoids at 50% probability.

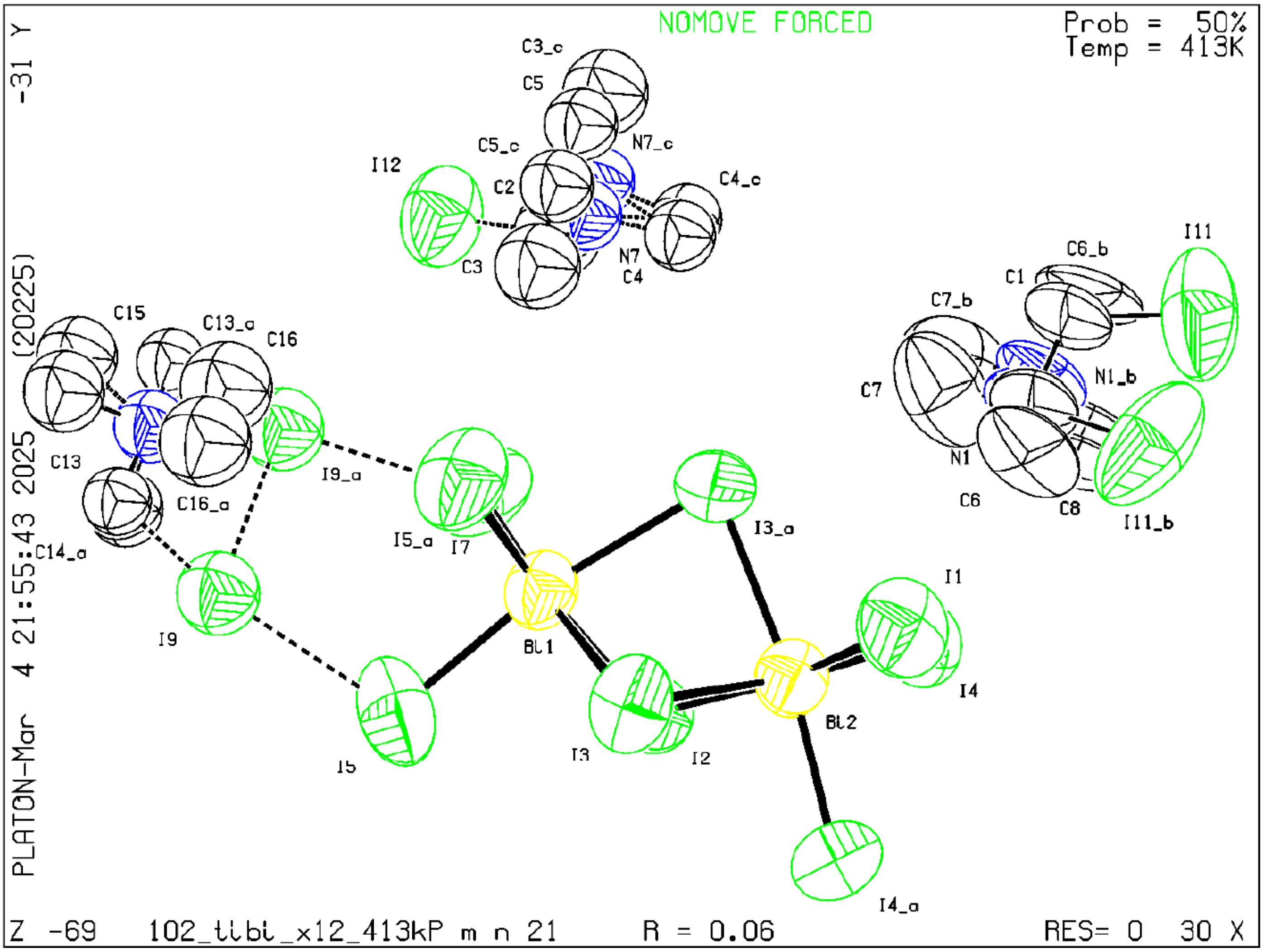

Figure S26: Thermal ellipsoid plot of $(TMIM)_3Bi_2I_9$ at 413K, ellipsoids at 50% probability.

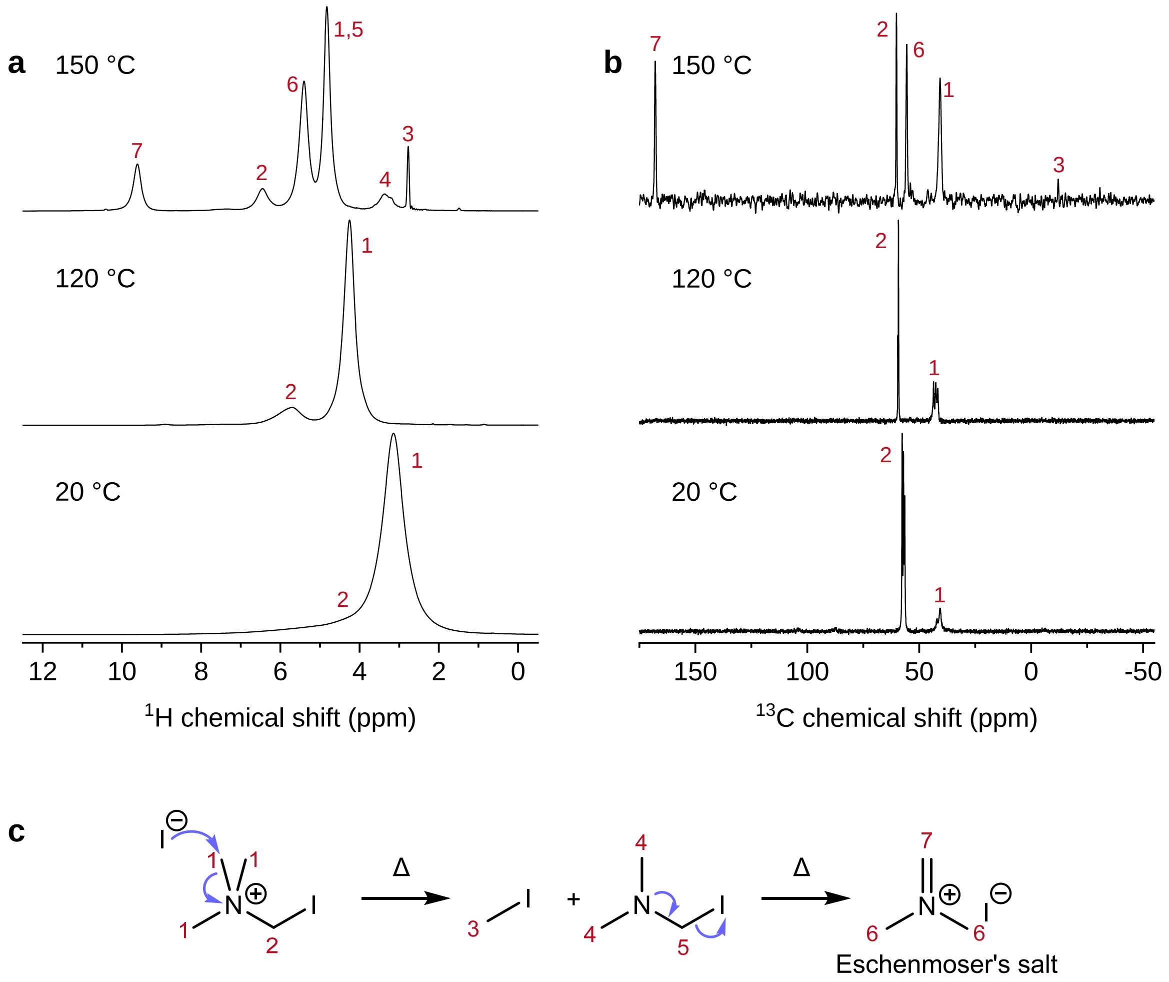

Figure S27: Additional NMR measurements. ^{1}H magic angle spinning (MAS) NMR (a) and ^{1}H-^{13}C cross-polarization MAS NMR (b) spectra of $(TMIM)_3Bi_2I_9$ at 20, 120 and 150 °C. Note that the varying efficacy of ^{1}H-^{13}C cross-polarization caused by the differing chemical environments corresponding to each signal shown in **b** mean that these spectra are not quantitative. In particular, rapid isotropic reorientation of iodomethane (labelled **3**) in the gas phase should lead to no ^{1}H-^{13}C cross-polarization signal; a small amount of CH_3I is nevertheless detected in the CP experiment suggesting that some CH_3I molecules are tightly bound in the solid state. **(c)** Mechanistic rationalization of pyrolysis of $(TMIM)^{+}I^{-}$ at elevated temperature. The mechanism is consistent with that originally proposed by Eschenmoser.[52]

Bibliography

(1) MacRae, C. F.; Sovago, I.; Cottrell, S. J.; Galek, P. T. A.; McCabe, P.; Pidcock, E.; Platings, M.; Shields, G. P.; Stevens, J. S.; Towler, M.; Wood, P. A. Mercury 4.0: From Visualization to Analysis, Design and Prediction. *J Appl Crystallogr* **2020**, *53* (1), 226–235. https://doi.org/10.1107/S1600576719014092.

(2) Winter, G.; Waterman, D. G.; Parkhurst, J. M.; Brewster, A. S.; Gildea, R. J.; Gerstel, M.; Fuentes-Montero, L.; Vollmar, M.; Michels-Clark, T.; Young, I. D.; Sauter, N. K.; Evans, G. DIALS: Implementation and Evaluation of a New Integration Package. *research papers Acta Cryst* **2018**, *74*, 85–97. https://doi.org/10.1107/S2059798317017235.

(3) Kratzert, D.; Holstein, J. J.; Krossing, I. DSR: Enhanced Modelling and Refinement of Disordered Structures with SHELXL. *J Appl Crystallogr* **2015**, *48* (3), 933–938. https://doi.org/10.1107/S1600576715005580/FS5104SUP1.PDF.

(4) Dolomanov, O. V.; Bourhis, L. J.; Gildea, R. J.; Howard, J. A. K.; Puschmann, H. OLEX2: A Complete Structure Solution, Refinement and Analysis Program. *J Appl Crystallogr* **2009**, *42* (2), 339–341. https://doi.org/10.1107/S0021889808042726.

(5) Sheldrick, G. M. Crystal Structure Refinement with SHELXL. *J Appl Crystallogr* **2015**, *71* (1), 3–8. https://doi.org/10.1107/S2053229614024218.

(6) Sheldrick, G. M. SHELXT – Integrated Space-Group and Crystal-Structure Determination. *J Appl Crystallogr* **2015**, *71* (1), 3–8. https://doi.org/10.1107/S2053273314026370.

(7) Sheldrick, G. M. A Short History of SHELX. *J Appl Crystallogr* **2007**, *64* (1), 112–122. https://doi.org/10.1107/S0108767307043930.

(8) Coelho, A. A. TOPAS and TOPAS-Academic: An Optimization Program Integrating Computer Algebra and Crystallographic Objects Written in C++. *J Appl Crystallogr* **2018**, *51* (1), 210–218. https://doi.org/10.1107/S1600576718000183.

(9) Purusottam, R. N.; Bodenhausen, G.; Tekely, P. Determination of Sample Temperature in Unstable Static Fields by Combining Solid-State 79Br and 13C NMR. *Journal of Magnetic Resonance* **2014**, *246*, 69–71. https://doi.org/10.1016/J.JMR.2014.06.021.

(10) O’Dell, L. A.; Schurko, R. W.; Harris, K. J.; Autschbach, J.; Ratcliffe, C. I. Interaction Tensors and Local Dynamics in Common Structural Motifs of Nitrogen: A Solid-State 14N NMR and DFT Study. *J Am Chem Soc* **2011**, *133* (3), 527–546. https://doi.org/10.1021/JA108181Y/SUPPL_FILE/JA108181Y_SI_001.PDF.

(11) Killgore, J. P.; Robins, L.; Collins, L. Electrostatically-Blind Quantitative Piezoresponse Force Microscopy Free of Distributed-Force Artifacts. *Nanoscale Adv* **2022**, *4* (8), 2036–2045. https://doi.org/10.1039/D2NA00046F.

(12) Nečas, D.; Klapetek, P. Gwyddion: An Open-Source Software for SPM Data Analysis. *Central European Journal of Physics* **2012**, *10* (1), 181–188. https://doi.org/10.2478/S11534-011-0096-2/MACHINEREADABLECITATION/RIS.

(13) Vrije Universiteit. ADF 2025.1, SCM, Theoretical Chemistry, . Amsterdam 2025.

(14) Baerends, E. J.; Aguirre, N. F.; Austin, N. D.; Autschbach, J.; Bickelhaupt, F. M.; Bulo, R.; Cappelli, C.; van Duin, A. C. T.; Egidi, F.; Fonseca Guerra, C.; Förster, A.; Franchini, M.; Goumans, T. P. M.; Heine, T.; Hellström, M.; Jacob, C. R.; Jensen, L.; Krykunov, M.; van Lenthe, E.; Michalak, A.; Mitoraj, M. M.; Neugebauer, J.; Nicu, V. P.; Philipsen, P.; Ramanantoanina, H.; Rüger, R.; Schreckenbach, G.; Stener, M.; Swart, M.; Thijssen, J. M.; Trnka, T.; Visscher, L.; Yakovlev, A.; van Gisbergen, S. The Amsterdam Modeling Suite. *Journal of Chemical Physics* **2025**, *162* (16), 162501. https://doi.org/10.1063/5.0258496/3344863.

(15) Gasevic, T.; Stückrath, J. B.; Grimme, S.; Bursch, M. Optimization of the R2SCAN-3c Composite Electronic-Structure Method for Use with Slater-Type Orbital Basis Sets. *Journal of Physical Chemistry A* **2022**, *126* (23), 3826–3838. https://doi.org/10.1021/ACS.JPCA.2C02951/ASSET/IMAGES/LARGE/JP2C02951_0017.JPEG.

(16) Giannozzi, P.; Baroni, S.; Bonini, N.; Calandra, M.; Car, R.; Cavazzoni, C.; Ceresoli, D.; Chiarotti, G. L.; Cococcioni, M.; Dabo, I.; Dal Corso, A.; De Gironcoli, S.; Fabris, S.; Fratesi, G.; Gebauer, R.; Gerstmann, U.; Gougoussis, C.; Kokalj, A.; Lazzeri, M.; Martin-Samos, L.; Marzari, N.; Mauri, F.; Mazzarello, R.; Paolini, S.; Pasquarello, A.; Paulatto, L.; Sbraccia, C.; Scandolo, S.; Sclauzero, G.; Seitsonen, A. P.; Smogunov, A.; Umari, P.; Wentzcovitch, R. M. QUANTUM ESPRESSO: A Modular and Open-Source Software Project for Quantumsimulations of Materials. *Journal of Physics: Condensed Matter* **2009**, *21* (39), 395502. https://doi.org/10.1088/0953-8984/21/39/395502.

(17) Giannozzi, P.; Andreussi, O.; Brumme, T.; Bunau, O.; Buongiorno Nardelli, M.; Calandra, M.; Car, R.; Cavazzoni, C.; Ceresoli, D.; Cococcioni, M.; Colonna, N.; Carnimeo, I.; Dal Corso, A.; De Gironcoli, S.; Delugas, P.; Distasio, R. A.; Ferretti, A.; Floris, A.; Fratesi, G.; Fugallo, G.; Gebauer, R.; Gerstmann, U.; Giustino, F.; Gorni, T.; Jia, J.; Kawamura, M.; Ko, H. Y.; Kokalj, A.; Kücükbenli, E.; Lazzeri, M.; Marsili, M.; Marzari, N.; Mauri, F.; Nguyen, N. L.; Nguyen, H. V.; Otero-De-La-Roza, A.; Paulatto, L.; Poncé, S.; Rocca, D.; Sabatini, R.; Santra, B.; Schlipf, M.; Seitsonen, A. P.; Smogunov, A.; Timrov, I.; Thonhauser, T.; Umari, P.; Vast, N.; Wu, X.; Baroni, S. Advanced Capabilities for Materials Modelling with Quantum ESPRESSO. *Journal of Physics: Condensed Matter* **2017**, *29* (46), 465901. https://doi.org/10.1088/1361-648X/AA8F79.

(18) Perdew, J. P.; Burke, K.; Ernzerhof, M. Generalized Gradient Approximation Made Simple. *Phys Rev Lett* **1996**, *77* (18), 3865. https://doi.org/10.1103/PhysRevLett.77.3865.

(19) Hamann, D. R. Optimized Norm-Conserving Vanderbilt Pseudopotentials. *Phys Rev B Condens Matter Mater Phys* **2013**, *88* (8), 085117. https://doi.org/10.1103/PHYSREVB.88.085117/83_BI.TXT.

(20) van Setten, M. J.; Giantomassi, M.; Bousquet, E.; Verstraete, M. J.; Hamann, D. R.; Gonze, X.; Rignanese, G. M. The PseudoDojo: Training and Grading a 85 Element Optimized Norm-Conserving Pseudopotential Table. *Comput Phys Commun* **2018**, *226*, 39–54. https://doi.org/10.1016/J.CPC.2018.01.012.

(21) Resta, R. Theory of the Electric Polarization in Crystals. *Ferroelectrics* **1992**, *136* (1), 51–55. https://doi.org/10.1080/00150199208016065;WGROUP:STRING:PUBLICATION.

(22) Resta, R. Macroscopic Polarization in Crystalline Dielectrics: The Geometric Phase Approach. *Rev Mod Phys* **1994**, *66* (3), 899. https://doi.org/10.1103/RevModPhys.66.899.

(23) King-Smith, R. D.; Vanderbilt, D. Theory of Polarization of Crystalline Solids. *Phys Rev B* **1993**, *47* (3), 1651. https://doi.org/10.1103/PhysRevB.47.1651.

(24) Vanderbilt, D. Berry-Phase Theory of Proper Piezoelectric Response. *Journal of Physics and Chemistry of Solids* **2000**, *61* (2), 147–151. https://doi.org/10.1016/S0022-3697(99)00273-5.

(25) Feldmann, C. Crystal Structure of Tris(Tetramethylammonium) Dibismuth Nonaiodide, [N(CH3)4]3Bi2I9. *Zeitschrift für Kristallographie - New Crystal Structures* **2001**, *216* (1–4), 487–488. https://doi.org/10.1524/NCRS.2001.216.14.487.

(26) Bernatowicz, P.; Kubica, D.; Ociepa, M.; Wodyński, A.; Gryff-Keller, A. Scalar Relaxation of the Second Kind. A Potential Source of Information on the Dynamics of Molecular Movements. 4. Molecules with Collinear C–H and C–Br Bonds. *Journal of Physical Chemistry A* **2014**, *118* (23), 4063–4070. https://doi.org/10.1021/JP5037298.

(27) Lyerla, J. R.; Grant, D. M.; Bertrand, R. D. Field-Dependent Contributions to Carbon-13 Nuclear Relaxation. *Journal of Physical Chemistry* **2002**, *75* (26), 3967–3971. https://doi.org/10.1021/J100695A009.
(28) Abragam, A. *The Principles of Nuclear Magnetism*; Clarendon Press, 1961.
(29) Shmyreva, A. A.; Safdari, M.; Furó, I.; Dvinskikh, S. V. NMR Longitudinal Relaxation Enhancement in Metal Halides by Heteronuclear Polarization Exchange during Magic-Angle Spinning. *J Chem Phys* **2016**, *144* (22). https://doi.org/10.1063/1.4953540.
(30) Dognon, J. P.; Pyykkö, P. Determining Nuclear Quadrupole Moments of Bi and Sb from Molecular Data. *Physical Chemistry Chemical Physics* **2023**, *25* (4), 2758–2761. https://doi.org/10.1039/D2CP04747K.
(31) *IEEE Xplore Full-Text PDF:* https://ieeexplore.ieee.org/stamp/stamp.jsp?tp=&arnumber=475550&tag=1 (accessed 2025-02-21).
(32) Gao, J.; Xue, D.; Liu, W.; Zhou, C.; Ren, X. Recent Progress on BaTiO3-Based Piezoelectric Ceramics for Actuator Applications. *Actuators 2017, Vol. 6, Page 24* **2017**, *6* (3), 24. https://doi.org/10.3390/ACT6030024.
(33) Rödel, J.; Jo, W.; Seifert, K. T. P.; Anton, E. M.; Granzow, T.; Damjanovic, D. Perspective on the Development of Lead-Free Piezoceramics. *Journal of the American Ceramic Society* **2009**, *92* (6), 1153–1177. https://doi.org/10.1111/J.1551-2916.2009.03061.X.
(34) Reznitchenko, L. A.; Turik, A. V.; Kuznetsova, E. M.; Sakhnenko, V. P. Piezoelectricity InNaNbO3 Ceramics. *Journal of Physics: Condensed Matter* **2001**, *13* (17), 3875. https://doi.org/10.1088/0953-8984/13/17/308.
(35) García-Zaldívar, O.; Escamilla-Díaz, T.; Ramírez-Cardona, M.; Hernández-Landaverde, M. A.; Ramírez-Bon, R.; Yañez-Limón, J. M.; Calderón-Piñar, F. Ferroelectric-Paraelectric Transition In A Membrane With Quenched-Induced δ-Phase Of PVDF. *Scientific Reports 2017 7:1* **2017**, *7* (1), 1–8. https://doi.org/10.1038/s41598-017-06044-y.
(36) Fu, D.-W.; Cai, H.-L.; Liu, Y.; Ye, Q.; Zhang, W.; Zhang, Y.; Chen, X.-Y.; Giovannetti, G.; Capone, M.; Li, J.; Xiong, R.-G. Diisopropylammonium Bromide Is a High-Temperature Molecular Ferroelectric Crystal. *Science (1979)* **2013**, *339* (6118), 425–428. https://doi.org/10.1126/science.1229675.
(37) You, Y. M.; Liao, W. Q.; Zhao, D.; Ye, H. Y.; Zhang, Y.; Zhou, Q.; Niu, X.; Wang, J.; Li, P. F.; Fu, D. W.; Wang, Z.; Gao, S.; Yang, K.; Liu, J. M.; Li, J.; Yan, Y.; Xiong, R. G. An Organic-Inorganic Perovskite Ferroelectric with Large Piezoelectric Response. *Science (1979)* **2017**, *357* (6348). https://doi.org/10.1126/science.aai8535.
(38) Liao, W. Q.; Tang, Y. Y.; Li, P. F.; You, Y. M.; Xiong, R. G. Large Piezoelectric Effect in a Lead-Free Molecular Ferroelectric Thin Film. *J Am Chem Soc* **2017**, *139* (49), 18071–18077. https://doi.org/10.1021/JACS.7B10449/SUPPL_FILE/JA7B10449_SI_003.CIF.
(39) Liao, W. Q.; Tang, Y. Y.; Li, P. F.; You, Y. M.; Xiong, R. G. Competitive Halogen Bond in the Molecular Ferroelectric with Large Piezoelectric Response. *J Am Chem Soc* **2018**, *140* (11), 3975–3980. https://doi.org/10.1021/JACS.7B12524/SUPPL_FILE/JA7B12524_SI_006.CIF.
(40) Liao, W. Q.; Zhao, D.; Tang, Y. Y.; Zhang, Y.; Li, P. F.; Shi, P. P.; Chen, X. G.; You, Y. M.; Xiong, R. G. A Molecular Perovskite Solid Solution with Piezoelectricity Stronger than Lead Zirconate Titanate. *Science (1979)* **2019**, *363* (6432). https://doi.org/10.1126/science.aav3057.
(41) Zhang, Z. X.; Zhang, H. Y.; Zhang, W.; Chen, X. G.; Wang, H.; Xiong, R. G. Organometallic-Based Hybrid Perovskite Piezoelectrics with a Narrow Band Gap. *J Am Chem Soc* **2020**, *142* (41). https://doi.org/10.1021/jacs.0c09288.

(42) Chen, X. G.; Song, X. J.; Zhang, Z. X.; Li, P. F.; Ge, J. Z.; Tang, Y. Y.; Gao, J. X.; Zhang, W. Y.; Fu, D. W.; You, Y. M.; Xiong, R. G. Two-Dimensional Layered Perovskite Ferroelectric with Giant Piezoelectric Voltage Coefficient. *J Am Chem Soc* **2020**, *142* (2), 1077–1082. https://doi.org/10.1021/JACS.9B12368/SUPPL_FILE/JA9B12368_SI_004.CIF.

(43) Guo, T. M.; Gong, Y. J.; Li, Z. G.; Liu, Y. M.; Li, W.; Li, Z. Y.; Bu, X. H. A New Hybrid Lead-Free Metal Halide Piezoelectric for Energy Harvesting and Human Motion Sensing. *Small* **2021**. https://doi.org/10.1002/smll.202103829.

(44) Ye, H. Y.; Tang, Y. Y.; Li, P. F.; Liao, W. Q.; Gao, J. X.; Hua, X. N.; Cai, H.; Shi, P. P.; You, Y. M.; Xiong, R. G. Metal-Free Three-Dimensional Perovskite Ferroelectrics. *Science (1979)* **2018**, *361* (6398), 151–155. https://doi.org/10.1126/SCIENCE.AAS9330/SUPPL_FILE/AAS9330-YE-SM.PDF.

(45) Wang, C. F.; Wang, N.; Liu, L.; Miao, L. P.; Ye, H. Y.; Zhang, Y.; Shi, C. Enantiomeric Hybrid High-Temperature Multiaxial Ferroelectrics with a Narrow Bandgap and High Piezoelectricity. *Chinese Chemical Letters* **2023**, *34* (8), 108051. https://doi.org/10.1016/J.CCLET.2022.108051.

(46) Tao, K.; Zhang, B.; Li, Q.; Yan, Q.; Tao, K.; Zhang, B.; Li, Q.; Yan, Q. Centimeter-Sized Piezoelectric Single Crystal of Chiral Bismuth-Based Hybrid Halide with Superior Electrostrictive Coefficient. *Small* **2023**, *19* (15), 2207663. https://doi.org/10.1002/SMLL.202207663.

(47) Zhang, Y. Z.; Sun, D. S.; Gao, J. X.; Hua, X. N.; Chen, X. G.; Mei, G. Q.; Liao, W. Q. A Semiconducting Organic–Inorganic Hybrid Perovskite-Type Non-Ferroelectric Piezoelectric with Excellent Piezoelectricity. *Chem Asian J* **2019**, *14* (7), 1028–1033. https://doi.org/10.1002/ASIA.201801921.

(48) Zhang, J.; Han, S.; Ji, C.; Zhang, W.; Wang, Y.; Tao, K.; Sun, Z.; Luo, J. [(CH3)3NH]3Bi2I9: A Polar Lead-Free Hybrid Perovskite-Like Material as a Potential Semiconducting Absorber. *Chemistry - A European Journal* **2017**, *23* (68), 17304–17310. https://doi.org/10.1002/CHEM.201703346;WEBSITE:WEBSITE:CHEMISTRY-EUROPE;REQUESTEDJOURNAL:JOURNAL:15213765;WGROUP:STRING:PUBLICATION.

(49) Deswal, S.; Panday, R.; Naphade, D. R.; Dixit, P.; Praveenkumar, B.; Zaręba, J. K.; Anthopoulos, T. D.; Ogale, S.; Boomishankar, R.; Deswal, S.; Panday, R.; Boomishankar, R.; Naphade, D. R.; Anthopoulos, T. D.; Zaręba, J. K.; Ogale, S. Efficient Piezoelectric Energy Harvesting from a Discrete Hybrid Bismuth Bromide Ferroelectric Templated by Phosphonium Cation. *Chemistry – A European Journal* **2022**, *28* (33), e202200751. https://doi.org/10.1002/CHEM.202200751.

(50) Sahoo, S.; Deka, N.; Boomishankar, R. Piezoelectric Energy Harvesting of a Bismuth Halide Perovskite Stabilised by Chiral Ammonium Cations. *CrystEngComm* **2022**, *24* (35), 6172–6177. https://doi.org/10.1039/D2CE00866A.

(51) Feldmann, C. Crystal Structure of Tris(Tetramethylammonium) Dibismuth Nonaiodide, [N(CH3)4]3Bi2I9. *Zeitschrift fur Kristallographie - New Crystal Structures* **2001**, *216* (1–4), 487–488. https://doi.org/10.1524/NCRS.2001.216.14.487/MACHINEREADABLECITATION/RIS.

(52) Schreiber, J.; Maag, H.; Hashimoto, N.; Eschenmoser, A. Dimethyl(Methylene)Ammonium Iodide. *Angewandte Chemie International Edition in English* **1971**, *10* (5), 330–331. https://doi.org/10.1002/ANIE.197103301.
